# HLA-A2.1-restricted ECM1-derived epitope LA through DC cross-activation priming CD8^+^ T and NK cells: a novel therapeutic tumour vaccine

**DOI:** 10.1186/s13045-021-01081-7

**Published:** 2021-04-28

**Authors:** Zhaojin Yu, Wensi Liu, Ying He, Mingli Sun, Jiankun Yu, Xue Jiao, Qiang Han, Haichao Tang, Bing Zhang, Yunkai Xian, Jing Qi, Jing Gong, Wang Xin, Gang Shi, Fengping Shan, Rui Zhang, Jianping Li, Minjie Wei

**Affiliations:** 1 Department of Pharmacology, School of Pharmacy, China Medical University, No. 13, Beihai Road, Dadong District, Shenyang, Liaoning Province China; 2 Liaoning Key Laboratory of Molecular Targeted Antitumour Drug Development and Evaluation, Liaoning Cancer Immune Peptide Drug Engineering Technology Research Centre, Key Laboratory of Precision Diagnosis and Treatment of Gastrointestinal Tumours, Ministry of Education, China Medical University, No.77, Puhe Road, Shenyang North New Area, Shenyang, Liaoning Province China; 3 Liaoning Medical Diagnosis and Treatment R&D Centre Co. Ltd., Shenyang, Liaoning Province China; 4 The Third Department of Medical Oncology, The Fourth Hospital of China Medical University, Shenyang City, Liaoning Province China; 5 Department of Colorectal Surgery, Cancer Hospital of China Medical University, Liaoning Cancer Hospital & Intitute, No.77, Xiaoheyan Road, Dadong District, Shenyang, Liaoning Province China; 6 Department of Immunology, College of Basic Medical Science, China Medical University, Shenyang, Liaoning Province China; 7 Transfusion Medicine Institute, Liaoning Blood Centre, Shenyang, Liaoning Province China; 8 Transfusion Medicine Institute, Harbin Blood Centre, Harbin, Heilongjiang Province China; 9 Department of Pharmacy, Harrison International Peace Hospital, Hengshui, Hebei Province China

**Keywords:** Therapeutic tumour vaccine, Extracellular matrix protein 1, CTL epitope, DC cross-presentation, DC-NK crosstalk

## Abstract

**Background:**

CD8^+^ T cell-mediated adaptive cellular immunity and natural killer (NK) cell-mediated innate immunity both play important roles in tumour immunity. This study aimed to develop therapeutic tumour vaccines based on double-activation of CD8^+^ T and NK cells.

**Methods:**

The immune Epitope database, Molecular Operating Environment software, and enzyme-linked immunosorbent assay were used for epitope identification. Flow cytometry, confocal microscopy, UPLC-QTOF-MS, and RNA-seq were utilized for evaluating immunity of PBMC-derived DCs, CD8^+^ T or NK cells and related pathways. *HLA-A2.1* transgenic mice combined with immunologically reconstituted tumour-bearing mice were used to examine the antitumour effect and safety of epitope vaccines.

**Results:**

We identified novel HLA-A2.1-restricted extracellular matrix protein 1(ECM1)-derived immunodominant epitopes in which LA induced a potent immune response. We also found that LA-loaded DCs upregulated the frequency of CD3^+^/CD8^+^ T cells, CD45RO^+^/CD69^+^ activated memory T cells, and CD3^−^/CD16^+^/CD56^+^ NK cells. We demonstrated cytotoxic granule release of LA/DC-CTLs or LA/DC-NK cells and cytotoxicity against tumour cells and microtissue blocks via the predominant IFN-γ/perforin/granzyme B cell death pathway. Further investigating the mechanism of LA-mediated CD8^+^ T activation, we found that LA could be internalized into DCs through phagocytosis and then formed a LA-MHC-I complex presented onto the DC surface for recognition of the T cell receptor to upregulate Zap70 phosphorylation levels to further activate CD8^+^ T cells by DC-CTL interactions. In addition, LA-mediated DC-NK crosstalk through stimulation of the TLR4-p38 MAPK pathway increased MICA/B expression on DCs to interact with NKG2D for NK activation. Promisingly, LA could activate CD8^+^ T cells and NK cells simultaneously via interacting with DCs to suppress tumours in vivo. Moreover, the safety of LA was confirmed.

**Conclusions:**

LA-induced immune antitumour activity through DC cross-activation with CD8^+^ T and NK cells, which demonstrated proof-of-concept evidence for the capability and safety of a novel therapeutic tumour vaccine.

**Supplementary Information:**

The online version contains supplementary material available at 10.1186/s13045-021-01081-7.

## Background

CD8^+^ T cells are critical for therapeutic tumour vaccines, activating the immune system to treat existing tumours or prevent tumour recurrence [1, 2]. Cytotoxic T lymphocyte (CTL) epitopes derived from tumour antigens provide an avenue for tumour-specific CD8^+^ T cell activation and have recently received considerable research attention. However, tumour progression is directly related not only to the tolerance of CD8^+^ T cells but also to that of natural killer (NK) cells. NK cells with distinct mechanisms of target cell recognition can exert broad antitumour effects without off-targets and are an important component of the innate immune system [3, 4]. Therefore, developing therapeutic tumour vaccines, especially through combination of the adaptive immune response of CD8^+^ T cells and innate immune response of NK cells to exert targeted antitumour effects is a promising strategy [5, 6].

Extracellular matrix protein 1 (ECM1), a classical tumour antigen [7, 8] with intrinsic characteristics of overexpression on tumour cell surface to facilitate the recognition of immune cells, could be a good target for therapeutic tumour vaccines. In the present study, we identified novel ECM1-derived HLA-A2.1 CTL epitopes and found LA and YL with potent DC (dendritic cell)-CTL antitumour effects. Surprisingly, LA could induce DC-NK antitumour effects as well. We performed functional assays on cell lines, patient-derived primary tumour cells, and microtissue blocks. We further conducted RNA-sequencing (RNA-seq)-based transcriptome analysis, and mouse model (xenograft mouse models and a allograft tumour-bearing HLA-A2.1 Tg mouse model)-based effect verification to identify mechanism of double-activation with DC-CTLs and DC-NK cells against tumours, which paved the way for the development of novel therapeutic tumour vaccines.

## Methods

### Ethics statement

This study was approved by the Ethics Committee of China Medical University (Shenyang, China; Approval No. [2016]054). All healthy volunteers and patients who provided clinical specimens signed the written informed consent form. The need for written informed consent was waived by the Ethics Committee for patients who provided clinical data, pathological tissue sections, and follow-up data due to the retrospective nature of the study. All procedures were performed in studies involving human participants were in accordance with 1964 Declaration of Helsinki principles and its later amendments or comparable ethical standards.

### Collection and synthesis of CTL epitopes

The HLA-A2.1-restricted CTL epitopes from the amino acid sequence of ECM1 (Table 1) were predicted using Immunological Epitope Database (IEDB) (http://www.iedb.org). All peptides, including LA (ECM1_9-17_, LVLTYLAVA) and YL (ECM1_43-51_, YAAPPSPPL), were synthesized by Chinese Peptide Co., Ltd. (Shanghai, China) with a purity of > 95% as determined by high-performance liquid chromatography (HPLC) (Additional file 1: Fig. S1a). The molecular weight of the peptide product was verified by mass spectrometry (Additional file 1: Fig. S1b). Besides, the endotoxin content of peptides was below 0.07 EU/mg obtained with the Limulus amebocyte lysate assay (photometric method) (Additional file 1: Fig. S1c).

**Table 1:** Identification of potential HLA-A2.1-restricted CTL epitopes from ECM1

Epitope	Start	End	Length	Sequence	IC_50_ (nM)^a^	*S* value^b^
NP	168	176	9	PPGRPSPDN	46,882.04	− 11.09
KA	90	98	9	KLLPAQLPA	15	− 36.26
YV	488	496	9	YLSPGDEQV	18.15	− 34.22
IV	409	417	9	ILTIDISRV	102.40	− 38.65
AV	8	16	9	ALVLTYLAV	174.81	− 39.46
YL	43	51	9	YAAPPSPPL	267.8	− 40.34
LA	9	17	9	LVLTYLAVA	334.48	− 42.45
QP	86	94	9	LQQEKLLPA	392.06	− 41.10

^a^IC_50_, the half maximal inhibitory concentration, obtained from IEDB, IC_50_ < 500 nM

^b^*S* value, the free-energy between docking molecules calculated by MOE

### Cell lines

Human non-triple-negative breast cancer cell line MCF-7 and human melanoma cell lines (SK-MEL-28 and A375) were cultured in the Dulbecco’s modified Eagle’s medium (DMEM; 12,800,017, Gibco, Gaithersburg, MD, USA) containing 10% foetal bovine serum (FBS; FND500, ExCell Bio, Inc., Shanghai, China); human triple-negative breast cancer cell line BT549 and human lung cancer cell line H2228 were cultivated in the Roswell Park Memorial Institute-1640 (RPMI-1640) medium (31,800,022, Gibco) with 10% FBS; human hepatocellular carcinoma cell line Hep G2 was cultured in the minimum essential medium (MEM) (12,500,062, Gibco), containing 10% FBS; human mammary epithelial cell line MCF-10A was cultivated in the MCF-10A complete medium (SCSP-575, The Cell Bank of Type Culture Collection, Chinese Academy of Sciences, Shanghai, China); T2 cells were cultured in the Iscove’s modified Dulbecco’s medium (IMDM) (12,440,053, Gibco) with 10% FBS. All the above-mentioned cells were cultured at 37 °C in presence of 5% CO_2_. Human triple-negative breast cancer cell line MDA-MB-231 and human colorectal cancer cell line SW480 were cultivated in L15 medium (41,300,039, Gibco) containing 10% FBS at 37 °C in air. All the cells were harvested in logarithmic growth phase. The sequencing-based typing (SBT) method was used for identification of HLA-A alleles at genomic level by Beijing Bo Furui Gene Diagnosis Technology Co., Ltd. (Beijing, China) (Additional file 1: Table S1).

### Animals

Mice were bred and housed under specific pathogen-free conditions at China Medical University. *HLA-A2.1* transgenic mice (8-week-old) were purchased from Jackson Laboratory (003475, Bar Harbor, ME, USA); Non-obese diabetic/severe combined immunodeficiency (NOD/SCID) mice (5-week-old, no. 406) were purchased from Beijing Vital River Laboratory Animal Technology Co., Ltd. (Beijing, China). All animal experiments were carried out in accordance with the guidelines published by the Institutional Animal Care and Use Committee of China Medical University.

### Induction of epitope/DC-CTL

CD8^+^ T-cells were purified (> 95%) from a cell suspension harvested from non-adherent peripheral blood mononuclear cells (PBMCs), using a CD8a^+^ T Cell Isolation Kit (130–045-201, Miltenyi Biotec, Bergisch Gladbach, Germany) according to the manufacturer’s instructions. CD8^+^ T cells were cultured in the ImmunoCult™-XF T Cell Expansion Medium (10,981, STEMCELL Technologies Inc., Vancouver, Canada) with ImmunoCult™ Human CD3/CD28/CD2 T Cell Activator (10,970, STEMCELL Technologies Inc.). DCs were resuspended in serum-free AIM-V medium (Gibco) and were stimulated with 50 μg/ml peptide twice with an interval of 24 h, treated with 30 ug/mL mitomycin C (A4452, APExBIO, Houston, TX, USA) for 30 min and washed. CD8^+^ T cells were stimulated with the above-described DCs at a ratio of 20: 1 on day 1 and day 6 to generate epitope/DC-CTL.

### Cytotoxicity assay of epitope/DC-CTL

Target cells were labelled with 25 uM calcein-AM (C326, DOJINDO, Kumamoto, Japan) for 25 min. The effector cells (epitope/DC-CTL) and target cells were cocultured at different ratios (E: T = 5:1, 10:1, 20:1, 40:1) for 4 h. The supernatant was detected by multimode reader (Tristar, Berlin, Germany) with an excitation wavelength of 485 nm and an emission wavelength of 535 nm. The assay was performed in triplicate. The percentage of specific lysis was calculated as follows: (OD of experimental release—OD of spontaneous release)/(OD of maximum release—OD of spontaneous release) × 100% (OD, optical density). The target cells of the spontaneous release group were cultured in the medium alone, and the target cells of the maximal release group were coincubated with 2% Triton X-100.

### Induction of epitope/DC-NK

NK cells were screened from PBMCs (DC-) by the Human NK Cell Isolation Kit (130–092-657, Miltenyi Biotec), and unlabelled cells were collected. NK cells were cultured in NK medium (DKW34-SCN1, Dakewe Biotech Co., Ltd., Shenzhen, China) and mixed with epitope-pulsed DCs at a ratio of 20:1 on day 1 and day 6 to generate epitope/DC-NK cells.

### Cytotoxicity assay of epitope/DC-NK

Epitope/DC-NK-mediated lysis of tumour cells was analysed using a lactate dehydrogenase (LDH) release cytotoxicity assay kit (Beyotime Institute of Biotechnology, Shanghai, China) at different ratios (E: T = 5:1, 10:1, 20:1, 40:1) according to the manufacturer's instructions. LDH release values were normalized to spontaneous LDH release by detecting effector cells in the same culture medium. The cytotoxicity was calculated as follows: Cytotoxicity (%) = [(OD of experimental release – OD of spontaneous release of effector cells – OD of spontaneous release of target cells)/(OD of maximum release of target cells– OD of spontaneous release of target cells)] × 100.

### In vivo study of immune effect

LA- or YL-ISA (Montanide ISA 51, SEPPIC, Paris, France) containing LA or YL was inoculated into inguinal region of *HLA-A2.1* transgenic mice for three times (day -13, day -7, day -1). Natural saline (NS), LA, YL, and ISA were used as control groups. Six days following the 3rd injection (day 5), DC and splenocyte phenotypes were detected.

#### In vivo study of inhibitory effects on the growth of the transplanted tumours

NOD/SCID mice received a subcutaneous injection of 2.5 × 10^6^ MDA-MB-231 cells, and then, they received 2 × 10^7^ splenocytes of *HLA-A2.1* transgenic mice intravenously. Splenocytes were injected three times, and the first immunization on HLA-A2.1 Tg mice started on day -13 and splenocytes were obtained on day 5. The second immunization started on day -10 and splenocytes were obtained on day 8, and the third immunization started on day -7 and splenocytes were obtained on day 11. Two batches of animals were included to assess the growing volume of xenograft tumours and the survival time. Paraffin-embedded sections of tumour tissues were prepared for immunohistochemistry (ICH). After staining with rabbit anti-CD8 alpha antibody (1:2000; ab217344, Abcam, Cambridge, UK) or NK1.1 monoclonal antibody (1:100; MA1-70,100, Carlsbad, CA, USA), the infiltration of CD8^+^ T and NK cells was detected. The tumours were measured in a blinded fashion.

#### In vivo study of inhibition of tumour metastasis

A metastasis model was established by tail vein injection of 2.5 × 10^6^ MDA-MB-231-luc cells in NOD/SCID mice. Then, those mice intravenously received 2 × 10^7^ splenocytes of *HLA-A2.1* transgenic for three times. In vivo fluorescence imaging was conducted on day 24. Lung tumour burden was assessed at the end of the experiment (day 25) by an in vivo imaging system and the organs were fixed. Lung metastatic tumour nodules were visually identified and further confirmed by haematoxylin and eosin (H&E) staining. Paraffin-embedded sections of lung tissue were prepared for IHC and stained with rabbit anti-CD8-α antibody or NK1.1 monoclonal antibody. Two batches of animals were included to assess the metastasis and the survival time.

#### Safety review of epitopes

The bone marrow aspiration was prepared from marrow fluids obtained from *HLA-A2.1* transgenic mice inoculated for 3 times and was then stained with Wright-Giemsa stain (BA4017, Baso Diagnostics Inc., Beijing, China). Liver, kidney, heart, lung, spleen, stomach, and intestine tissue sections were stained with H&E. Blood samples were tested in parallel by a haematology analyser. Liver function indexes and renal function indexes, such as aspartate aminotransferase (AST), alanine aminotransferase (ALT), uric acid (UA), and creatinine (CRE), were measured by a biochemical analyser. The whole body and main organs were weighed to calculate the organ indexes.

#### HLA-2.1/ECM1-overexpressing transplanted tumour model

Female *HLA-A2.1* transgenic mice (6-week-old) received a subcutaneous injection of 1 × 10^6^ ECM1^+^/HLA-A_2_^+^ E0771 into their right forelimbs. The mice were randomly assigned into the following groups: 1) control, 2) ISA, 3) YL-ISA, 4) LA-ISA, 5) ISA + TAK242, 6) YL-ISA + TAK242, and 7) LA-ISA + TAK242. TAK-242 (ethyl (6R)-6-[N-(2-chloro-4-fluorophenyl)sulfamoyl]cyclohex-1-ene-1-carboxylate; A3850, APExBio) is a novel small molecule that selectively inhibits Toll-like receptor 4 (TLR4) [9]. Human TLR4 is homologous to mouse TLR4, and TAK-242 possesses similar inhibitory effects on mouse and human monocytes [10]. TAK242 diluent in normal saline was injected into *HLA-A2.1* transgenic mice at a dose of 1 mg/kg 3 h before epitope-ISA vaccination. The dose of LA or YL was 1 mg/20 g body weight. ISA, LA-ISA or YL-ISA were inoculated into the inguinal region of *HLA-A2.1* transgenic mice in the corresponding groups on day 2, day 8, and day 14. Two batches of animals were included to evaluate the growing volume of transplanted tumours and the survival time. Paraffin-embedded sections of tumour tissue were prepared for IHC. The tumours were measured in a blinded fashion.

#### Statistical analysis

The SPSS 22.0 software (IBM, Armonk, NY, USA) was used to perform statistical analysis. ECM1 expression data, which were abnormally distributed, were compared between two groups using the Wilcoxon signed-ranks test. Categorical data were compared with the Pearson’s chi-square test or the Fisher’s exact test. Survival probabilities were estimated by the Kaplan–Meier method and assessed by the log-rank test. Other results were expressed as mean ± standard deviation (SD) and were compared with the independent samples t-tests. Probability values (*P*) < 0.05 was considered statistically significant.

See further methods in supplementary materials.

## Results

### Identification of HLA-A2.1-restricted ECM1-derived immunodominant CTL epitopes

**Fig. 1:**
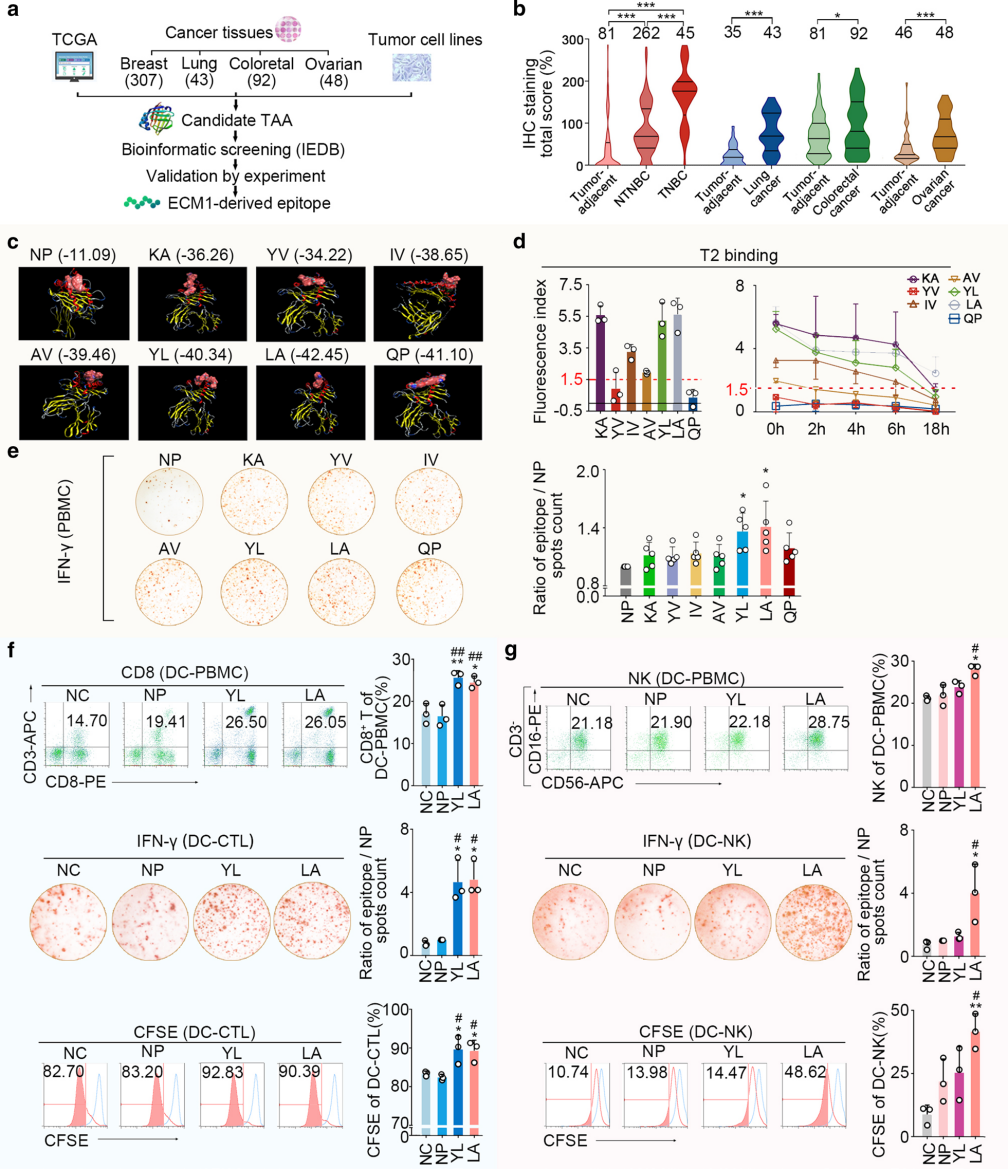
Identification of HLA-A2.1-restricted ECM1-derived epitopes with a double activation of CD8^+^ T and NK cells. **a** Strategy of identifying CTL epitopes. **b** ECM1 expression increased significantly in various tumour tissues compared with tumour-adjacent tissues by immunohistochemistry. **c** The combination between peptides and HLA-A2.1 by MOE. The number represents the S value, the free-energy between docking molecules. **d** T2 binding assay. Left, HLA-A2.1-binding affinity of epitopes (*n* = 3). T2 cells were cultured with peptide (50 μg/mL) and β2-microglobulin (3 μg/ml) for 18 h. Fluorescence index (FI) > 1.5, high affinity. Right, HLA-A2-binding stability of epitopes (*n* = 3). T2 cells were stimulated with peptide for 18 h and then treated with brefeldin A (10 μg/mL) for 0, 2, 4, 6 and 18 h, respectively. DC_50_ > 6 h, high stability. DC_50_, the time point when the mean FI value at 0 h was reduced to 50%. **e** After stimulation with peptides (50 μg/mL), IFN-γ secretion by rhECM1-sensitized (10 μg/mL) HLA-A2.1^+^ PBMC was detected by ELISPOT (*n* = 5). **f** Frequency of CD8^+^ T cells in HLA-A2.1^+^ PBMC (DC-) stimulated with peptide-pulsed DCs (*n* = 3). IFN-γ secretion and proliferative effect of epitope/DC-CTLs were detected by ELISPOT and CFSE assays (*n* = 3). **g** Frequency of NK cells (CD3^−^CD16^+^CD56^+^) in HLA-A2.1^+^ PBMC (DC-) stimulated with peptide-pulsed DCs (*n* = 3). IFN-γ secretion and proliferative effect of epitope/DC-NK cells were detected by ELISPOT and CFSE assays (*n* = 3). CFSE, carboxyfluorescein succinimidyl ester; NC, negative control, water; NP, negative peptide, the peptide (PPGRPSPDN) derived from ECM1 with the largest predicted IC_50_. **b** *P* values were obtained with Wilcoxon signed-rank test; error bars denote medians and interquartile ranges; ^*^P < 0.05, ^***^P < 0.001. **e–g** *P* values were obtained with independent-samples t-test; error bars denote standard deviation (SD); **e** Compared with NP, ^*^P < 0.05; **f**, **g** Compared with NC, ^*^P < 0.05, ^**^P < 0.01; compared with NP, ^#^*P* < 0.05, ^##^*P* < 0.01

We confirmed the rationality of ECM1 as a tumour-associated antigen with a high expression level in multiple tumours, such as breast cancer, especially in triple-negative breast cancer, by analysing data collected from The Cancer Genome Atlas (TCGA) database (Additional file 1: Fig. S2a), clinical tissue specimens and the corresponding clinical data (Fig. 1b, Additional file 1: Fig. S2b-f), and high ECM1 expression was closely correlated to tumour biology behaviour (Additional file 1: Tables S2, S3). We employed IEDB database [11] and MOE software to obtain seven ECM1-derived HLA-A2.1-restricted CTL epitopes, including KA, YV, IV, AV, YL, LA, and QP (Fig. 1c, Table 1). Further studies showed that LA and YL could stably bind to the HLA-A2.1 molecule (Fig. 1d) and significantly stimulated interferon γ (IFN-γ) secretion by ECM1 protein-sensitized HLA-A2.1^+^ PBMCs (Fig. 1e). Moreover, DCs loaded with LA or YL could significantly increase the frequencies of CD8^+^ T cell in PBMCs (Fig. 1f). Additionally, IFN-γ secretion and proliferation of CD8^+^ T cells were also significantly enhanced with the stimulation of LA- or YL-loaded DCs (Fig. 1f). These results indicated that both LA and YL could be used as ECM1-derived dominant CTL epitopes and promote the activation of CD8^+^ T cells.

In addition, we surprisingly found LA-loaded DCs (LA/DCs) could increase the NK cell population in PBMCs (Fig. 1g, Additional file 1: Fig. S2g, h). IFN-γ secretion and proliferation of LA/DC-stimulated NK cells were significantly increased (Fig. 1g). These results showed that LA/DC could activate CD8^+^ T and NK cells simultaneously, which was obviously different from the traditional CTL epitope with activation of only CD8^+^ T cells.

### LA-loaded DCs induced double antitumour effects by activation of CD8^+^ T and NK cells

We further evaluated the LA-loaded DCs induced double antitumour effects via activating CD8^+^ T and NK cells.

**Fig. 2:**
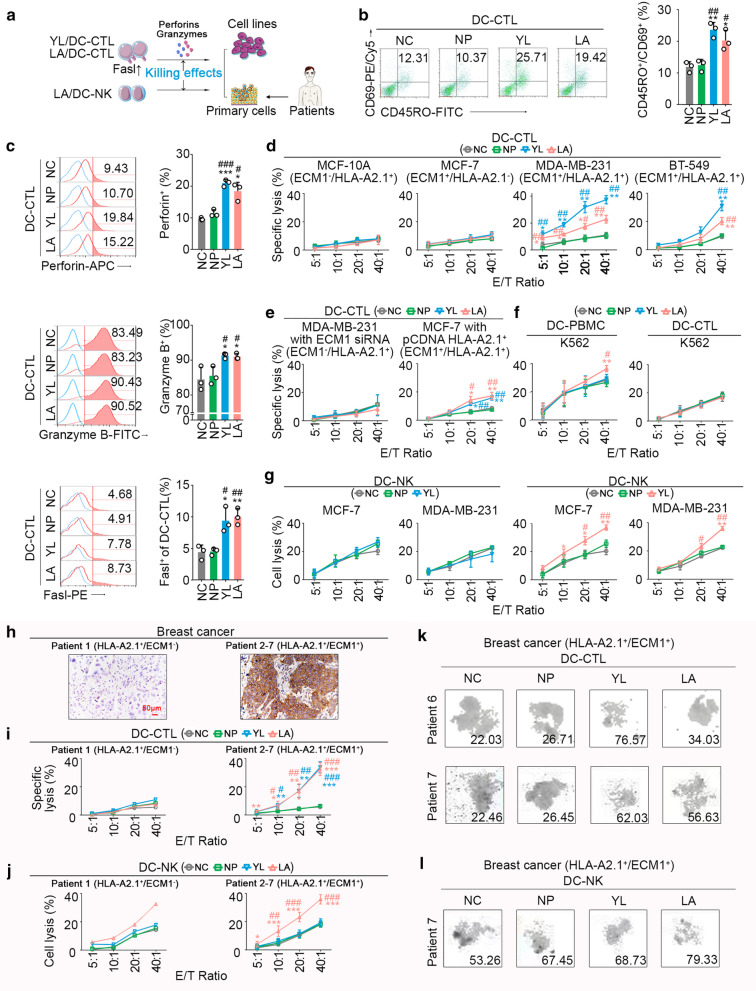
LA-pulsed DCs induced dual cytotoxicity of CTLs and NK cells against breast cancer in vitro. **a** Strategy of exploring cytotoxicity of YL/DC-CTL, LA/DC-CTL or LA/DC-NK. DCs were stimulated with 50 μg/ml peptide twice at an interval of 24 h, treated with 30 ug/mL mitomycin C for 30 min, and then cocultured with CD8^+^ T cells at a ratio of 1:20 on day 1 and day 6 to generate epitope/DC-CTLs. NK cells were cocultured with epitope-pulsed DCs at a ratio of 20:1 on day 1 and day 6 to generate epitope/DC-NK. **b** CD45RO and CD69 expression were significantly upregulated in YL/DC-CTLs or LA/DC-CTLs (*n* = 3), suggesting a stronger memory function. **c** Perforin/granzyme-B/FasL levels in YL/DC-CTLs or LA/DC-CTLs were significantly increased against MDA-MB-231 cells (HLA-A2.1^+^/ECM1^+^) using flow cytometry. **d** The cytotoxicity of epitope/DC-CTLs against epithelial cell MCF-10A (HLA-A2.1^+^/ECM1^−^), cancer cell MCF-7 (HLA-A2.1^−^/ECM1^+^), MDA-MB-231 (HLA-A2.1^+^/ECM1^+^), and BT-549 (HLA-A2.1^+^/ECM1^+^) was detected by calcein-release assay (*n* = 3); E/T ratio, effector/target cell ratio. **e** Cytotoxicity of epitope/DC-CTLs against ECM1-silenced MDA-MB-231 and HLA-A2.1-knocked-in MCF-7 cells (*n* = 3) **f** Cytotoxicity of DC-PBMCs or DC-CTLs against NK-sensitive K562 cells (*n* = 3). **g** Cytotoxicity of epitope/DC-NK cells against tumour cells (*n* = 3). **h** Representative immunohistochemical staining of ECM1. Scale bars, 50 μm. **i**, **j** Cytotoxicity of epitope/DC-CTLs or epitope/DC-NK cells on primary breast cancer cells from a HLA-A2.1^+^/ECM1^−^ patient (*n* = 1) and HLA-A2.1^+^/ECM1^+^ patients (*n* = 6). **k**, **l** Cytotoxicity of epitope/DC-CTLs (*n* = 2) or epitope/DC-NK cells(*n* = 1) on breast cancer microtissue blocks from HLA-A2.1^+^/ECM1^+^ patients. The number represented the inhibition rates (IR%). NC, negative control, water. NP, negative peptide, the peptide (PPGRPSPDN) derived from ECM1 with largest predicted IC_50_ of affinity with HLA-A2.1. **b–g**, **i**, **j** *P* values were obtained from independent-samples t-test; error bars denote standard deviation (SD). Compared with NC, ^*^P < 0.05, ^**^P < 0.01, ^***^P < 0.001; compared with NP, ^#^*P* < 0.05, ^##^*P* < 0.01, ^###^*P* < 0.001

First, we analysed antitumour effects of LA/DC-CTL. Phenotypically, we observed increased CD69^+^/CD45RO^+^ (activated memory) T cells after LA/DC stimulation on CD8^+^ T cells (Fig. 2b). Furthermore, LA/DC-CTLs showed effective antitumour responses, including FasL upregulation and increased secretion of perforin and granzyme B (Fig. 2c, Additional file 1: Fig. S3a). Meanwhile, LA/DC-CTL could exert significant cytotoxicity against HLA-A2.1^+^/ECM1^+^ breast cancer cell lines (MDA-MB-231 and BT-549), while no notable cytotoxicity on HLA-A2.1^+^/ECM1^−^ breast epithelial cell line MCF-10A or HLA-A2.1^−^/ECM1^+^ breast cancer cell line MCF-7 was detected (Fig. 2d, Additional file 1: Figs. S2f, S3b). Furthermore, there was no difference in cytotoxicity against MDA-MB-231 cells with ECM1 knockdown compared to the control group (Additional file 1: Fig. S3c, Fig. 2e), and the cytotoxicity was significantly elevated against HLA-A2.1 knock-in MCF-7 cells (Additional file 1: Fig. S3d, Fig. 2e). The results demonstrated that LA/DC-CTL could induce immune responses against tumours in an HLA-A2-restricted and ECM1-specific manner. Similarly, we found that LA/DC-CTL could generate remarkable cytotoxicity against HLA-A2.1^+^/ECM1^+^ primary cancer cells and microtissue blocks derived from fresh clinical specimens (Fig. 2h, i, k, Additional file 1: Fig. S4). YL/DC-CTL showed similar antitumour effects as well. Importantly, LA/DC-CTL and YL/DC-CTL did not exert noticeable cytotoxicity against HLA-A2.1^+^ PBMCs from healthy volunteers (Additional file 1: Fig. S5a).

We further attempted to indicate whether LA/DCs could enhance the immune function of NK cells (LA/DC-NK) against tumour cells. We found that LA/DC stimulation could significantly increase the cytotoxicity of PBMCs against the NK-sensitive K562 cell line, rather than the cytotoxicity of CTLs against K562 (Fig. 2f). We thus speculated that the enhanced cytotoxicity of PBMCs against K562 cells could be due to NK cell activation. Further study revealed that LA/DC-NK cells could generate significant cytotoxicity without HLA-A2.1 restriction against MDA-MB-231 (HLA-A2.1^+^) and MCF-7 (HLA-A2.1^−^) cells compared with the control group (Fig. 2g), whereas YL/DC could not enhance immune response of NK cells (Fig. 2g). Similarly, LA/DC-NK cells, rather than YL/DC-NK cells, could also generate remarkable cytotoxicity without restriction of ECM1 expression against primary cancer cells (Fig. 2h, j, l, Additional file 1: Fig. S4). In addition, LA/DC-NK cells did not produce cytotoxicity against PBMCs or DCs from healthy volunteers (Additional file 1: Fig. S5b, c).

### LA-mediated DC-CTL interaction to activate CD8^+^ T cells

**Fig. 3:**
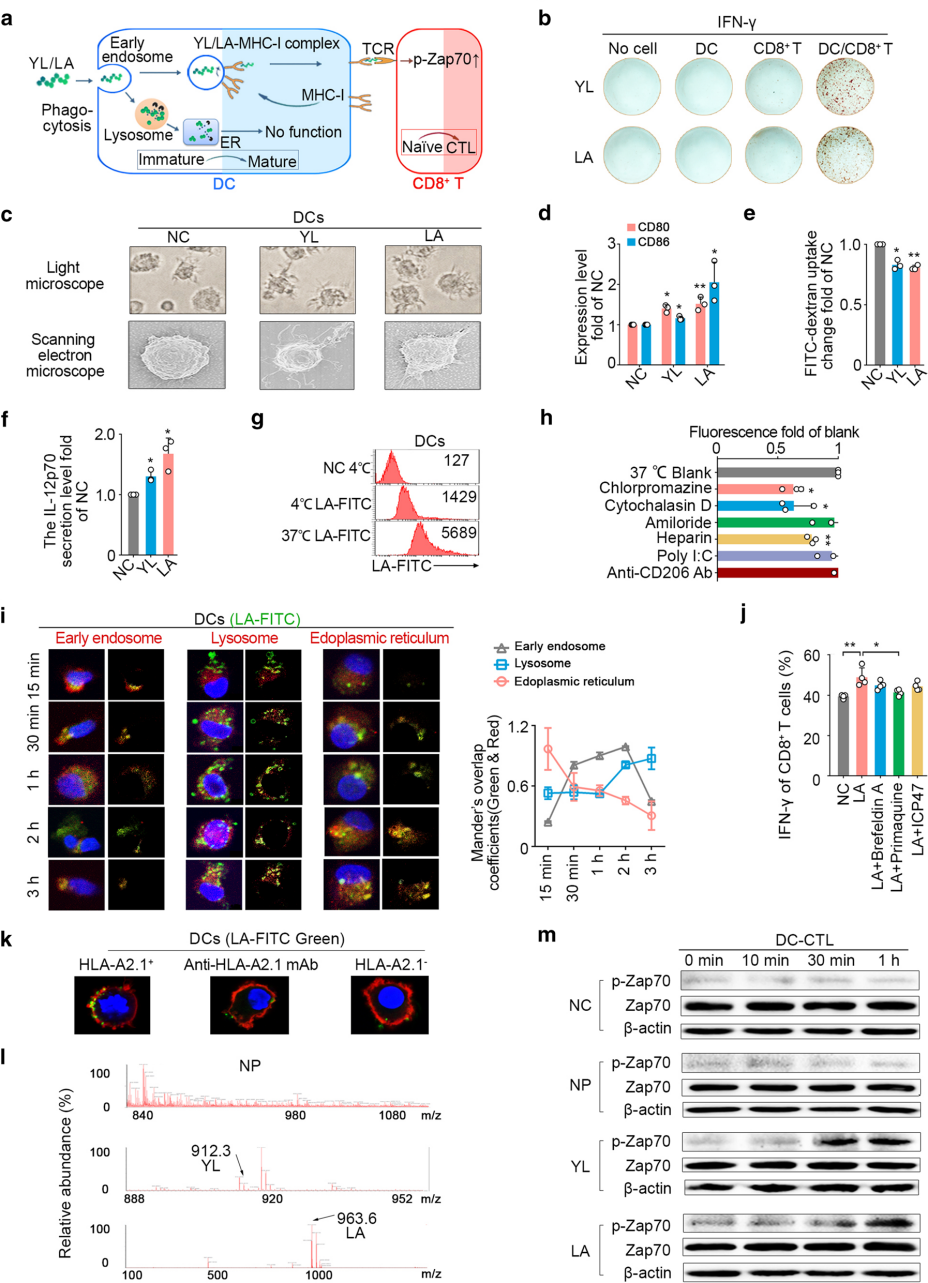
Mechanisms of DC-mediated cross-presentation of LA. **a** Pathway diagram of DC cross-presenting epitopes. **b** IFN-γ secretion by ELISPOT assay (*n* = 3). **c–f** Monocytes were obtained with a monocyte attachment medium (C-28051, PromoCell) from PBMCs and then cultured in DC generation medium A (C21050, PromoCell) to harvest immature DCs. Immature DCs were treated with 50 μg/ml peptide twice with an interval of 24 h. Maturation of YL/LA-pulsed DCs was promoted (*n* = 3). **c** Morphological examination. **d** Presentation ability. **e** Uptake capacity. **f** IL-12p70 secretion by ELISA. **g** LA was mainly taken up by immature DCs through an active transport (37 °C), and others might directly bind to DCs (4 °C). **h** LA was mainly taken up by phagocytosis (inhibited with cytochalasin D), partly by receptor-mediated internalization (inhibited with heparin) (*n* = 3). **i** Immunofluorescence analysis of co-localization between LA (green, FITC) and different subcellular compartments (red, early endosome, lysosome or endoplasmic reticulum) in immature DCs (blue, DAPI, nuclear) (*n* = 3). **j** The trace of LA was mainly concentrated on MHC-I molecular internalization (inhibited with primaquine), rather than vesicle secretion (inhibited with brefeldin A) and TAP transport (inhibited with ICP47) in DCs (*n* = 3). **k** The combination of LA-FITC and HLA-A2.1 on DC surface (blue, DAPI, nuclear; red, Dil, membrane; green, LA-FITC). **l** The presentation of LA (963.6) or YL (912.3) on DC surface by UPLC-QTOF-MS. **m** Immunoblot analysis of total Zap70 proteins and phosphorylated (p-) Zap70 in whole-cell lysates of epitope/DC-CTLs (*n* = 3). NC, negative control, water. NP, negative peptide, the peptide (PPGRPSPDN) derived from ECM1 with largest predicted IC_50_ of affinity with HLA-A2.1. **d–f**, **h**, **j** *P* values were obtained from independent-samples t-test; error bars denote standard deviation (SD). **d–f** Compared with NC, ^*^P < 0.05, ^**^P < 0.01; **h** Compared with 37 °C blank, ^*^P < 0.05, ^**^P < 0.01; **j** ^*^P < 0.05, ^**^P < 0.01

DCs are such an essential factor involved in CTL epitope activating CD8^+^ T cells (Fig. 3b). However, it is important to indicate how such exogenous LA could elicit CD8^+^ T cells through an endogenous major histocompatibility complex (MHC) class I (MHC-I) presentation pathway in DCs.

Mature state of DCs enables them to cross-process and present exogenous antigens, resulting in the activation of CD8^+^ T cells. After treatment with LA, the morphology of DCs tended to mature (Fig. 3c), the expression of B7 costimulatory molecules (CD80/86) was upregulated (Fig. 3d), phagocytosis of glucan decreased (Fig. 3e), and the secretion level of IL-12p70 was elevated (Fig. 3f). These results demonstrated that LA could promoted the maturation status of DCs.

We also found that LA could be phagocytized by DCs via active transport at 37 °C (Fig. 3g). Clathrin-mediated internalized inhibitor (chlorpromazine), phagocytosis inhibitor (cytochalasin D), and receptor-mediated uptake inhibitor (heparin) could significantly inhibit the uptake of LA into DCs, while neither scavenger receptor-mediated internalization inhibitor nor mannose receptor-mediated internalization inhibitor decreased the phagocytosis of LA into DCs (Fig. 3h). The confocal microscopy displayed the process of LA transport into DCs involved in early endosomes, lysosomes, endoplasmic reticulum in turn (Fig. 3i). The IFN-γ secretion of CD8^+^ T cells stimulated with LA-loaded DCs exhibited no noticeable difference when blocking the function of vesicle secretion or transporters associated with antigen processing (TAP), while significantly decreased when blocking the internalization function of DC surface molecules (Fig. 3j). We further incubated HLA-A2.1^+^ DCs with LA-FITC, and the results showed that the FITC fluorescence signals could be detected on HLA-A2.1^+^ DC surface, whereas they decreased and even disappeared on HLA-A2-blocked DCs or HLA-A2.1^−^ DCs (Fig. 3k). Additionally, we analysed the membrane proteins of LA-loaded DCs to identify the presence of LA by ultra-performance liquid chromatography-quadrupole time-of-flight mass spectrometry (UPLC-QTOF-MS) (Fig. 3l), which indirectly confirmed that LA could be presented on the DC surface in the form of binding to HLA-A2.1 molecules.

CTL epitope-HLA-A2.1 molecule complex needs to be recognized by T cell receptor (TCR) to trigger downstream signals for CD8^+^ T cell activation. We found that LA-loaded DCs significantly increased the phosphorylation level of Zap70, a key molecule in the TCR signalling pathway, in CD8^+^ T cells compared with the control group (Fig. 3m).

### LA-activated NK cells via DC-NK crosstalk

**Fig. 4:**
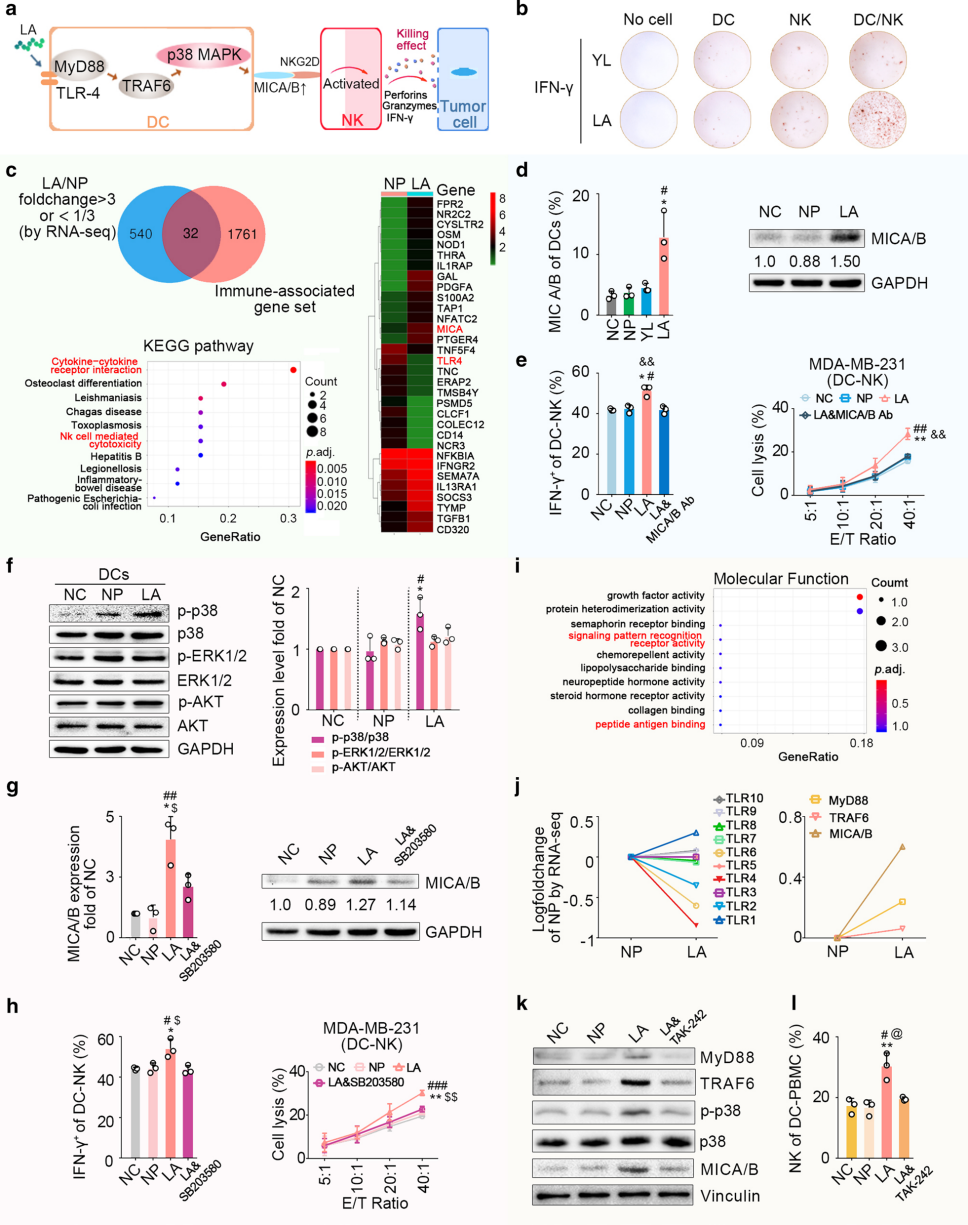
Mechanisms of LA-induced activation of NK cells. **a** LA-induced NK cell activation through MICA/B-NKG2D signalling between DCs and NK cells. **b** IFN-γ secretion of different cells stimulated with YL or LA using an ELISPOT assay (*n* = 3). **c** Immune-related differentially expressed genes between LA-pulsed DCs and NP-pulsed DCs by RNA-seq. **d** The MICA/B expression level in LA-pulsed DCs was significantly increased (*n* = 3). Left, flow cytometry. Right, western blot. **e** IFN-γ expression and cytotoxicity of NK cells were significantly elevated with stimulation with LA/DCs. MICA/B antibody (Ab) could block LA/DC-mediated NK activation. (*n* = 3). **f** Phosphorylation level of p38 was significantly upregulated in LA-pulsed DCs, rather than the phosphorylation level of ERK1/2 or AKT (*n* = 3). **g** SB203580 (10 μM), an inhibitor of the p38 MAPK pathway, blocked LA-mediated upregulation of MICA/B on DCs (*n* = 3). **h** SB203580 blocked LA/DC-mediated IFN-γ expression and NK cell cytotoxicity (*n* = 3). **i** The RNA-seq results showed the molecular functions of immune-related differentially expressed genes between LA-pulsed DCs and NP-pulsed DCs. **j** Left panel, TLR4 on LA-pulsed DCs decreased among TLRs by RNA-seq. Right panel, upregulation of TLR4 downstream signals (MYD88 and TRAF6) and MICA/B in LA-pulsed DCs by RNA-seq. **k** TAK242 (DCs were pretreated for 1 h with TAK-242, 10 μM) inhibited LA-mediated upregulation of MYD88, TRAF6, p38 phosphorylation, and MICA/B in DCs (*n* = 3). **l** LA/DC&TAK242 could not promote proliferation of NK cells in PBMCs (*n* = 3). NC, negative control, water. NP, negative peptide, the peptide (PPGRPSPDN) derived from ECM1 with largest predicted IC_50_ of affinity with HLA-A2.1. **d**, **e–h**, **l** *P* values were obtained from independent-samples t-test; error bars denote standard deviation (SD). Compared with NC, ^*^P < 0.05, ^**^P < 0.01; compared with NP, ^#^*P* < 0.05, ^##^*P* < 0.01, ^###^*P* < 0.001; compared with LA&MICA/B Ab, ^&&^*P* < 0.01; compared with LA&SB203580, ^$^*P* < 0.05, ^$$^*P* < 0.01; compared with LA&TAK242, ^@^*P* < 0.05

In the process of exploring the mechanism of LA-induced NK activation, we found that DCs play a key role (Fig. 4b). Hence, we initially investigated differences between LA-pulsed DCs and NP-pulsed DCs by RNA-seq-based transcriptome analysis (Fig. 4c). The results revealed that among the differentially expressed genes (FC > 3 or FC < 1/3), MHC class I chain-related A (MICA) was the most relevant molecule with NK activation. Among the differentially expressed genes, 32 immune-related genes were mainly enriched in cytokine-cytokine receptor interaction, NK cell-mediated cytotoxicity, etc. Flow cytometry and western blot (WB) assays further confirmed that LA could significantly upregulate the expression level of MICA/B in DCs (Fig. 4d). We also found that LA/DC-induced NK cells could significantly produce periforin, granzyme-B, and IFN-γ. However, LA-pulsed DCs pretreated with MICA/B monoclonal antibody could not significantly stimulate the secretion of these effectors by NK cells (Fig. 4e left, Additional file 1: Fig. S6a, b) and the cytotoxicity of NK cells against breast cancer cells (Fig. 4e right). These findings indicated that upregulation of MICA/B expression in DCs was critical in mediating the antitumour effects of LA/DC-NK cells.

Further research indicated that LA could upregulate the phosphorylation level of p38 in DCs to activate the p38 mitogen-activated protein kinase (MAPK) pathway, which is involved in regulating MICA/B [12], while it did not affect the MICA/B-related extracellular signal-regulated kinase (ERK)/MAPK pathway [13] or PI3K/Akt pathway [14] (Fig. 4f). Meanwhile, SB203580, an inhibitor of p38 MAPK pathway, blocked the increased MICA/B expression of LA-pulsed DCs (Fig. 4g), and the increased secretion of perforin, granzyme-B, IFN-γ, as well as cytotoxicity of LA/DC-induced NK cells against breast cancer (Fig. 4h). These results showed that LA could regulate MICA/B expression in DCs via the p38 MAPK pathway to further activate NK cells.

RNA-seq-based transcriptome analysis also indicated that immune-related differentially expressed genes of DCs with or without LA treatment mainly enriched in the following molecular functions: signalling pattern recognition receptor activity, peptide antigen binding, etc. (Fig. 4i). In the group of DCs treated with LA, TLR4 expression showed a pronounced downregulation trend among the TLRs (Fig. 4j left), and the expressions of TLR4 downstream signals, such as myeloid differentiation factor 88 (MyD88) and tumour necrosis factor receptor-associated factor 6 (TRAF6), were markedly upregulated (Fig. 4j right). TLR4 also showed the highest affinity with LA among TLRs using MOE (Additional file 1: Fig. S6c). We thus hypothesized that LA might be used as a TLR4 agonist. WB assays confirmed that LA could significantly upregulate the expression levels of MYD88 and TRAF6 in DCs (Fig. 4k). With further analysis of the relationship between TLR4 and LA-induced upregulation of p38 phosphorylation level, we found that in DCs treated with TAK242 (a specific inhibitor of TLR4 signalling), the upregulated expression of MYD88 and TRAF6, the increased expression levels of p38 phosphorylation and MICA/B (Fig. 4k), and the promoted NK proliferation of NK cells induced by LA all disappeared (Fig. 4l). These indicated that LA could trigger TLR4-related signals to stimulate the TLR4-p38 MAPK pathway in DCs to further upregulate MICA/B expression for NK cell activation (Fig. 4a).

### LA induced activation of CD8^+^ T and NK cells in ***HLA-A2.1*** transgenic mice

**Fig. 5:**
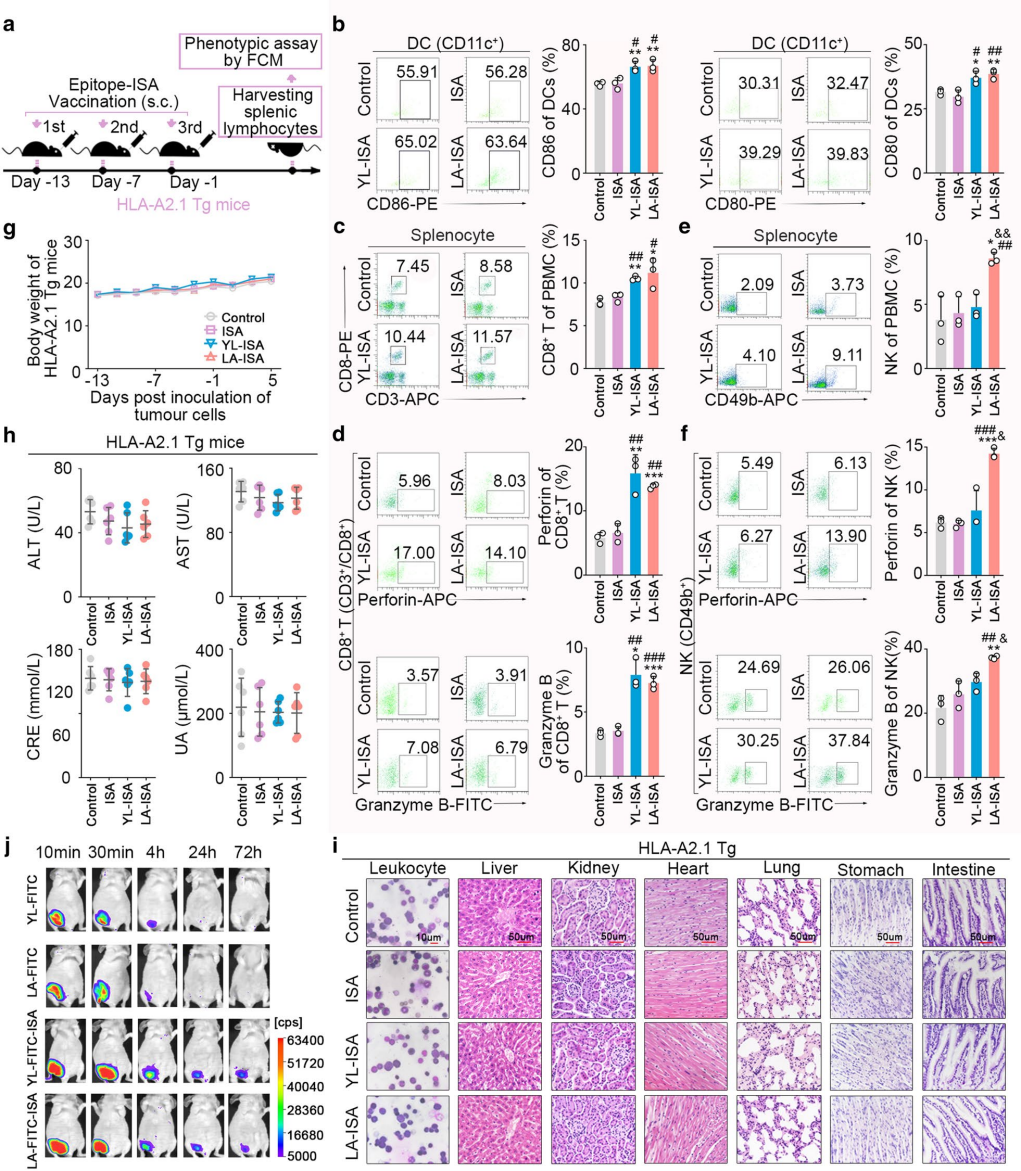
LA-ISA immunization induces responses of DC, CD8^+^ T, and NK cells in *HLA-A2.1* transgenic mice. **a** *HLA-A2.1* transgenic mice were immunized subcutaneously for three times with the same volume of YL-ISA, LA-ISA, ISA, or control. The dose of LA or YL was 1 mg/20 g body weight. After final immunization, splenocytes or peripheral blood cells were harvested and analysed by flow cytometry. **b** DC phenotype of peripheral blood cells (*n* = 3). **c** The frequency of CD8^+^ T cells in splenocytes (*n* = 3). **d** The expression levels of perforin and granzymes-B in CD8^+^ T cells. **e** The frequency of NK cells in splenocytes (*n* = 3). **f** The expression levels of perforin and granzymes-B in NK cells (*n* = 3). **g** No significant difference could be detected in body weight of *HLA-A2.1* transgenic mice immunized subcutaneously with YL-ISA, LA-ISA, ISA, or control (*n* = 6). **h** No significant difference was found in liver function (ALT, AST) and kidney function (CRE, UA) of *HLA-A2.1* transgenic mice immunized subcutaneously with YL-ISA, LA-ISA, ISA, or control (*n* = 6). **i** No significant difference was identified in pathological examination of vital organs of *HLA-A2.1* transgenic mice immunized subcutaneously with YL-ISA, LA-ISA, ISA, or control. Leukocytes (Wright Giemsa staining, scale bars, 10 μm). Liver, kidney, heart, lung, stomach, intestine (H&E staining, scale bars, 50 μm). **j** The stability of YL-FITC-ISA or LA-FITC-ISA in mice was detected periodically by an *in-vivo* imaging system. Control, *HLA-A2.1* transgenic mice were treated with normal saline. ISA, *HLA-A2.1* transgenic mice were treated with ISA. YL-ISA, *HLA-A2.1* transgenic mice were treated with YL-ISA. LA-ISA, *HLA-A2.1* transgenic mice were treated with LA-ISA. **b–f** *P* values were obtained with independent-samples t-test; error bars denote standard deviation (SD). Compared with control, ^*^P < 0.05, ^**^P < 0.01, ^***^P < 0.001. Compared with ISA, ^#^*P* < 0.05, ^##^*P* < 0.01, ^###^*P* < 0.001. Compared with YL-ISA, ^&^*P* < 0.05, ^&&^*P* < 0.01

To investigate whether LA could induce immune responses of DC-CTLs and DC-NK cells in vivo, the adjuvant ISA-51, which could improve the stability of peptides in vivo, was used to formulate peptide-ISA (Fig. 5j). *HLA-A2.1* transgenic mice were immunized with subcutaneously administration of peptide-ISA for three times (Fig. 5a). It was revealed that the expression levels of DC maturation markers (CD80 and CD86) were significantly elevated in peripheral blood DCs (CD11c^+^) of *HLA-A2.1* transgenic mice immunized with LA-ISA (Fig. 5b). Meanwhile, the frequencies of CD8^+^ T and NK cells (Fig. 5c, e) and the secretions of perforin and granzyme B in CD8^+^ T and NK cells (Fig. 5d, f) were significantly increased, while the frequency of CD4^+^ T cells did not significantly differ (Additional file 1: Fig. S7). In addition, consistent with the in vitro results, YL-ISA-immunized *HLA-A2.1* Tg mice only showed DC and CD8^+^ T cell priming.

We also evaluated the safety of epitope-ISA used to immunized *HLA-A2.1* transgenic mice. Among mice that were immunized with LA-ISA, YL-ISA, ISA, or NS, no significant difference was detected in their the bodyweight (Fig. 5g), haematological indexes of the peripheral blood (Additional file 1: Table S4), morphology of bone marrow cells (Fig. 5i), histological examination of vital organs (e.g. liver, kidney, heart, lung, stomach, and intestine) (Fig. 5i), visceral indexes of heart, lung, spleen, thoracic gland and brain (Additional file 1: Table S5), and functional indexes of liver and kidney (Fig. 5h).

### LA-sensitized effector cell immunological reconstitution in tumour-bearing NOD/SCID mice to exert anti-breast cancer effects

**Fig. 6:**
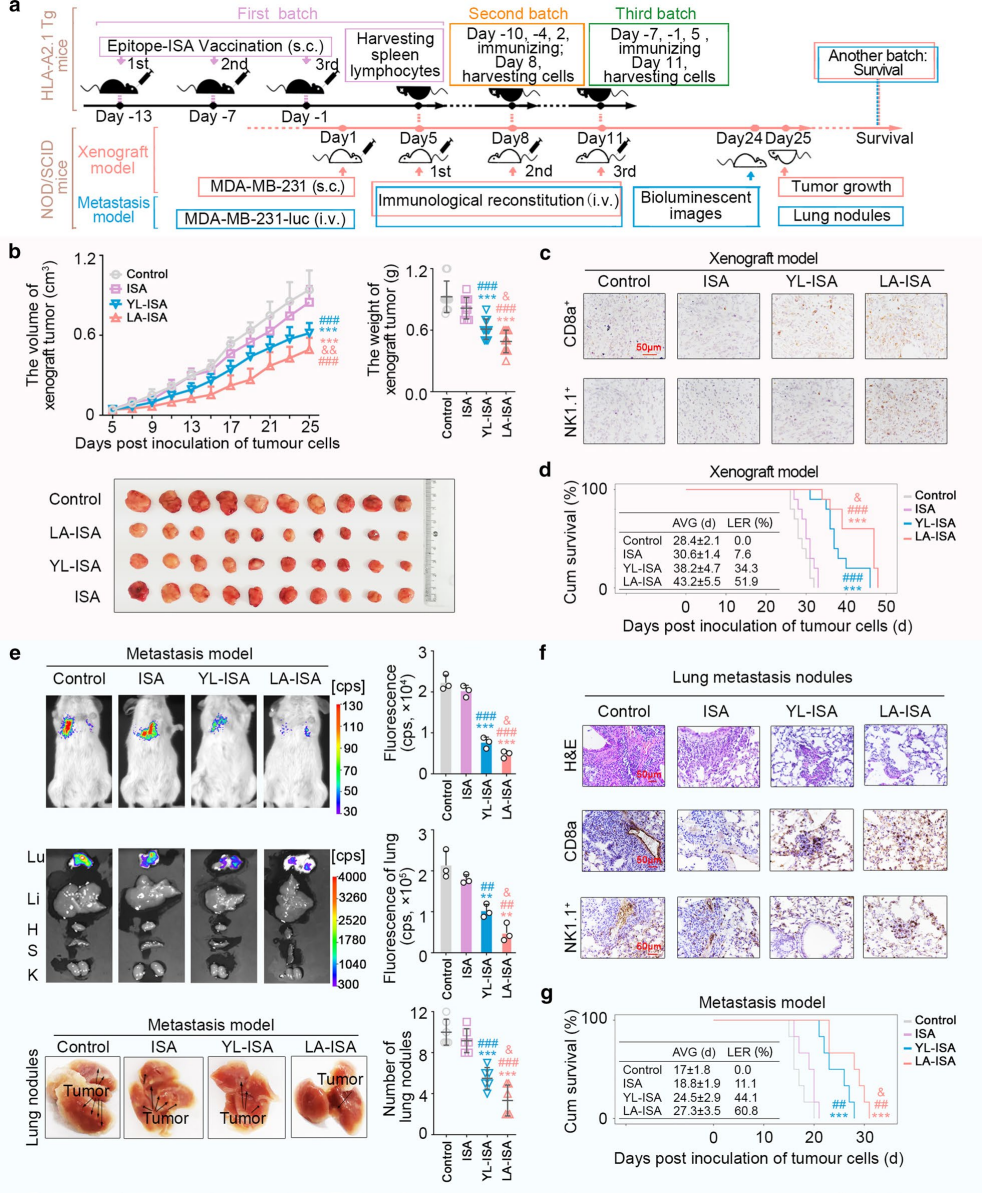
LA-ISA immunized spleen lymphocytes from H*LA-A2.1* transgenic mice against MDA-MB-231 cell-bearing NOD/SCID mice. **a** Immunological reconstruction pattern diagram. **b** Immunological reconstitution in tumour-bearing NOD-SCID mice was constructed using splenic lymphocytes harvested from *HLA-A2.1* transgenic mice immunized subcutaneously with YL-ISA, LA-ISA, ISA, or control (1 mg epitope/20 g body weight). Growth curve and weight of transplanted tumour at day 25 after tumour cell inoculation (*n* = 10). **c** Representative immunohistochemical staining of CD8a and NK1.1 in subcutaneously transplanted tumour tissues. **d** Survival (*n* = 10). AVG, average; LER (%), life extension rate. **e** Immunological reconstitution in a metastatic NOD/SCID mouse model was constructed using splenocytes harvested from *HLA-A2.1* transgenic mice immunized subcutaneously with YL-ISA, LA-ISA, ISA, or control (1 mg epitope/20 g body weight). Representative bioluminescence images and quantitative data of mice and vital organs (*n* = 3). Representative macroscopic images and quantitative data of pulmonary metastases (*n* = 6). Lu, lung; Li, liver; H, heart; S, spleen; K, kidney. **f** H&E staining of pulmonary metastases. Immunohistochemical staining of CD8a and NK1.1 in pulmonary metastases. **g** Survival (*n* = 6). AVG, average; LER (%), life extension rate. **c**, **f** Scale bars, 50 μm. Control, NOD/SCID mice inoculated with splenocytes from *HLA-A2.1* transgenic mice treated with normal saline. ISA, NOD/SCID mice inoculated with splenocytes from *HLA-A2.1* transgenic mice treated with ISA. YL-ISA, NOD/SCID mice inoculated with splenocytes from *HLA-A2.1* transgenic mice treated with YL-ISA. LA-ISA, NOD/SCID mice inoculated with splenocytes from *HLA-A2.1* transgenic mice treated with LA-ISA. **b**, **e** *P* values were obtained with independent-samples t-test; error bars denote standard deviation (SD); **d**, **g** *P* values were calculated by the log-rank test; **b**, **d**, **e**, **g** Compared with control, ^**^P < 0.01, ^***^P < 0.001; compared with ISA, ^##^*P* < 0.01, ^###^*P* < 0.01; compared with YL-ISA, ^&^*P* < 0.05, ^&&^*P* < 0.01

To investigate whether effector cells induced by LA-ISA could exert anti-breast cancer effects in vivo, NOD/SCID mice (recipient mice) transplanted with human breast cancer cells were inoculated intravenously with splenocytes from the LA-ISA-immunized *HLA-A2.1* transgenic mice (donor mice) for immunological reconstitution (Fig. 6a). Tumour progression in the LA-ISA group was significantly inhibited compared with that in the NS group (Fig. 6b), and the survival time of NOD/SCID mice was also significantly elongated in the LA-ISA group (Fig. 6d). The tumour volume was slightly reduced in the ISA-treated group, and the results showed no significant difference compared with the control group (*P* = 0.136, Fig. 6b). Furthermore, we constructed a MDA-MB-231-luc-inoculated NOD/SCID mouse model via injection subcutaneously into receptor mice (Fig. 6a). The results showed that the formation and proliferation of pulmonary metastases were inhibited in the LA-ISA group (Fig. 6e), and the survival time of mice was significantly elongated (Fig. 6g). The results confirmed the anti-breast cancer effects of splenocytes from LA-ISA-immunized *HLA-A2.1* transgenic mice in vivo. In addition, the safety of LA-induced splenocytes for immunological reconstitution mice was detected and there was no significant abnormity (Additional file 1: Fig. S8).

Further analysis showed that the antitumour effects of splenocytes induced by YL-ISA were markedly weaker compared with induction by LA-ISA in vivo (Fig. 6b, e). Histological examination showed that infiltration of CD8^+^ T cells in the transplanted tumour tissues (Fig. 6c) and pulmonary metastases (Fig. 6f) were significantly increased after immune reconstruction with YL-ISA- or LA-ISA-induced splenocytes compared with the control group. In contrast, the increased infiltration of NK cells only appeared in the LA-ISA group (Fig. 6c, g), which was consistent with the results that LA-ISA induced stronger antitumour effects than YL-ISA in vivo. This could be attributed to double-activation of CD8^+^ T and NK cells.

### LA exerted anti-breast cancer effects on HLA-2.1/ECM1 overexpressed allograft tumour-bearing *HLA-A2.1* transgenic mice

**Fig. 7:**
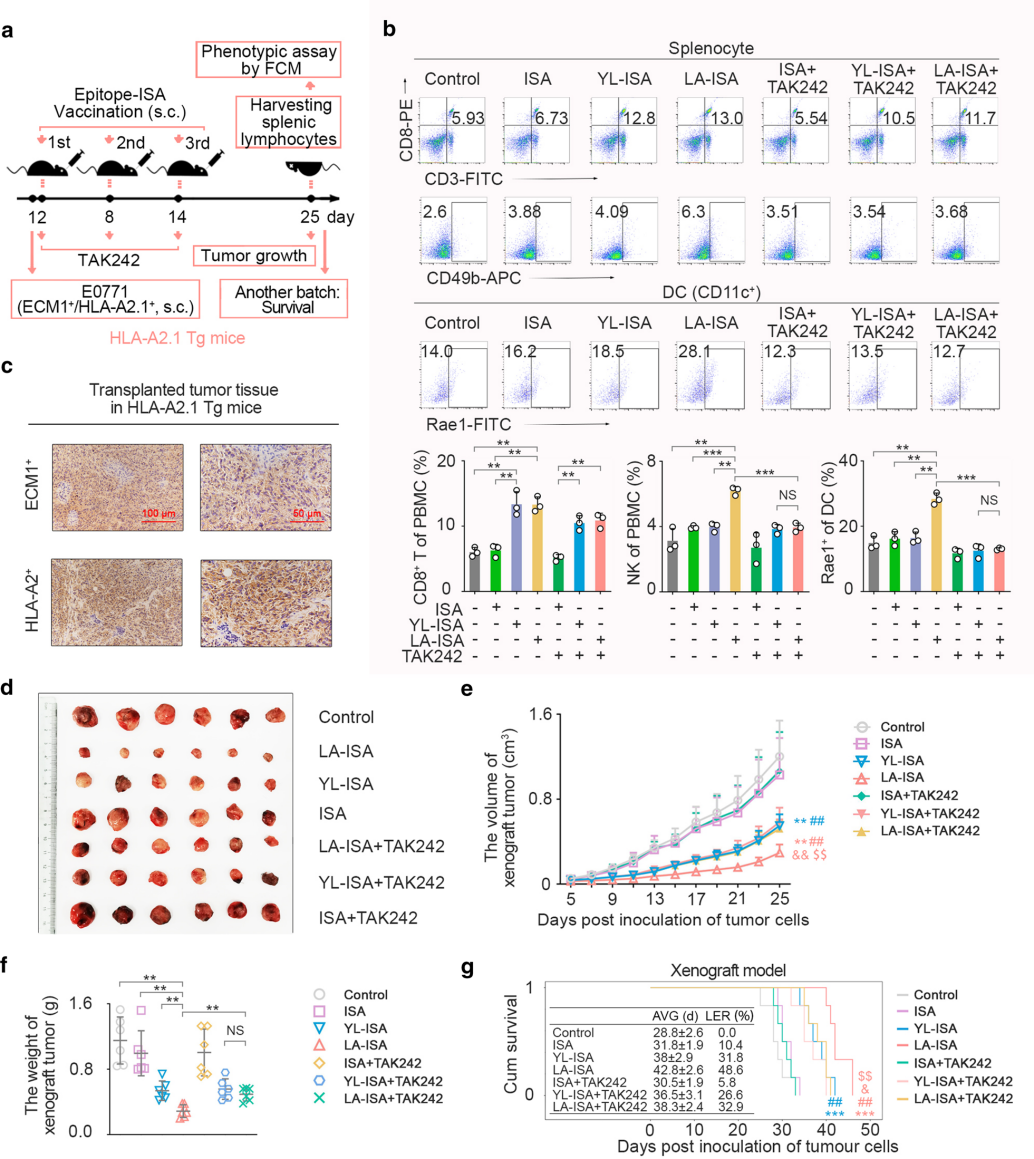
LA-ISA vaccination induces antitumour immune activities in HLA-A2.1^+^/ECM1^+^ allograft tumour-bearing *HLA-A2.1* transgenic mice. **a** Epitope vaccination pattern diagram. *HLA-A2.1* and human *ECM1* double-knock-in mouse mammary cancer cell line (E0771) was subcutaneously injected into *HLA-A2.1* transgenic mice to construct the allograft tumour model. LA- or YL-ISA was inoculated into *HLA-A2.1* transgenic mice for three times. The dose of LA or YL was 1 mg/20 g body weight. TAK242 diluent in normal saline was injected intravenously into *HLA-A2.1* transgenic mice at a dose of 1 mg/kg 3 h before epitope-ISA vaccination. **b** The frequencies of CD8^+^ T and NK cells (CD49b^+^) in splenocytes as well as the expression level of Rae1 in DCs from *HLA-A2.1* transgenic mice were detected by flow cytometry (*n* = 3). **c** Immunohistochemistry assay of ECM1 and HLA-A2 in tumour tissues. Scale bars, left panel, 100 μm, right panel, 50 μm. **d** The transplanted tumours were removed surgically at day 25 post-tumour implantation. **e** Growth curve of transplanted HLA-A2.1^+^/ECM1^+^ murine tumours (*n* = 6). **f** The weight of transplanted HLA-A2.1^+^/ECM1^+^ murine tumours at day 25 after tumour cell inoculation (*n* = 6). **g** Survival (*n* = 6). AVG, average; LER (%), life extension rate. Control, transgenic mice inoculated with normal saline. **b**, **e**, **f** *P* values were obtained with independent-samples t-test; error bars denote standard deviation (SD). **b**, **f** ^**^P < 0.01, ^***^P < 0.001, NS, not significant. **e** Compared with control, ^**^P < 0.01; compared with ISA, ^##^*P* < 0.01; compared with YL-ISA, ^&&^*P* < 0.01; compared with LA-ISA + TAK242, ^$$^*P* < 0.01. **g** *P* values calculated by the log-rank test; compared with control, ^***^P < 0.001; compared with ISA, ^##^*P* < 0.01; compared with YL-ISA, ^&^*P* < 0.05; Compared with LA-ISA + TAK242, ^$$^*P* < 0.01

*HLA-A2.1* and human *ECM1* double-knock-in mouse mammary cancer cell line (E0771) was subcutaneously injected in *HLA-A2.1* transgenic mice to construct an allograft tumour model for investigating the immune activation and the antitumour effects induced by LA under the same condition (Fig. 7a). Tumour progression in mice immunized with LA-ISA was inhibited significantly compared with the NC group or ISA group (Fig. 7d-f), and the survival time of mice was also significantly elongated in the LA-ISA group (Fig. 7g). Moreover, LA-ISA-induced immunity appeared with higher efficiencies than YL-ISA. These results were consistent with our findings achieved from the xenograft mouse model.

To further validate LA-induced NK activation by TLR4, we applied TAK-242 to inhibit TLR4 signalling (Fig. 7b). The results showed that the upregulated expression level of Rae1 (a ligand for mouse NKG2D with a very similar structure to MICA/B) in DCs and the increased frequency of NK cells from LA-ISA-immunized were impaired by TAK242. In contrast, the increased frequency of CD8^+^ T cells was noticeably elevated in the LA-ISA and YL-ISA groups with or without TAK242 treatment. These findings demonstrated that TAK-242 could significantly weaken the LA-ISA-induced antitumour effect of NK cells, rather than that of CD8^+^ T cells in vivo. Taken together, the results confirmed LA-induced NK cell cytolytic activity via TLR4 activation.

## Discussion

Although the recent emergence of epitopes from mutated neoantigens [15], mass spectrometry profiling of HLA-associated peptidomes [16], and antigens associated with peptide processing [17] have attracted extensive attention. These personalized epitope vaccines [18] are likely more expensive than developing a conventional vaccine based on tumour-specific antigens or tumour-associated antigens (TAAs). Therefore, TAA-derived CTL epitope vaccines may foster the clinical development of antitumour vaccines. ECM1 is overexpressed in multiple tumours, enabling ECM1-based epitopes to be broadly applied in diverse types of cancer. Additionally, the high expression level of ECM1 in skin may cause a strong immunity and even induce Lichen sclerosis [19, 20]; thus, ECM1, as a target, may elicit strong immune response rather than self-tolerance. Collectively, ECM1 is a more attractive immunotherapeutic target. We confirmed the feasibility of ECM1 as a universal TAA, identified the predominant HLA-A2.1-restricted epitopes derived from ECM1 (LA and YL), and verified their immunogenicity to induce DCs to elicit CD8^+^ T cells and potent cytotoxicity against breast cancer in vitro and in vivo. Therefore, LA and YL showed promising clinical potential as candidate predominant CTL epitopes. Additionally, we found that LA could induce not only a strong CTL antitumour immunity, but also an NK cells’ immune response, which was different from previous studies. Both adaptive and innate immune responses of LA were enhanced and LA presented a stronger antitumour effect than YL. Meanwhile, LA/DC-NK-induced toxic killing ability could escape the restriction of the expression levels of MHC-I and ECM1. Tumour cells with the loss of MHC-I molecules could escape CTL-mediated cytotoxicity [21], while could be susceptible to NK cell cytotoxicity [22]. Hence, LA-induced NK activation could compensate for the deficiency of CTL cytotoxicity. Our previous study showed that the LT peptide could induce antitumour effect of NK cells [23], rather than CTLs. The current study revealed that LA could induce the double-activation of CD8^+^ T and NK cells, which was not reported in previous researches.

The study revealed that LA could cross CD8^+^ T cells through MHC-I presentation pathway in DCs to induce CD8^+^ T cells, which was consistent with the classical pathway of cross-presentation of exogenous peptides [24]. Exogenous antigens are typically associated with immature DCs in vivo, and it is essential to promote DCs to tend to mature states for further cross-presentation [25]. We found that LA-ISA could stimulate immature DCs towards maturation and exhibited a favourable DC immunogenicity.

LA could be taken up by phagocytosis- and receptor-mediated internalization into DCs by active transport, which was consistent with pathway of classical exogenous antigen uptake of DCs [26]. The phagosome-to-cytosol pathway and vacuolar pathway are major mechanisms of cross-presenting antigens [24]. The current study confirmed that vacuolar pathway contributed more to cross-priming to LA in DCs than phagosome-to-cytosol pathway. The evidence for this comes from the fact that LA could be internalized into phagosomes, secreted by vesicles, reached lysosomes, and transferred into the endoplasmic reticulum by TAP. However, these epitopes, which are involved in the phagosome-to-cytosol pathway, could not mediate DC cross-priming. In addition, the confocal microscopy and UPLC-QTOF-MS displayed LA in form of a LA/MHC-I complex presented on DC surface. Although LA could bind directly to the HLA-A2 molecule on DC surface, the possibility of directly binding to the HLA molecule on cell surface is limited [27]. This may be because the majority of DCs are in an immature state with insufficient expression of MHC-I molecules in vivo [28]; thus, the intracellular presentation pathway is the main approach for LA to induce immunity [29–31]. The results further confirmed that the LA/MHC-I complex on DC surface could activate the TCR of CD8^+^ T cells to upregulate the phosphorylation level of ZAP70, a key downstream signal of the CD3-ζ chain [32]. Previous studies on cross-presentation of exogenous antigens by DCs mainly concentrated on microorganisms (such viruses), proteins, and long peptides [33–35], rather than short peptides. The current study depicted the mechanistic routine of short peptides represented by LA via DC cross-presentation.

We, for the first time, identified that DCs were necessary for LA to induce the NK cells immune response in the process of exploring the mechanism by which LA activates NK cells. Previous studies have shown a cross-talk occurs between DCs and NK cells. A number of contact-dependent factors could promote DC-NK cross-talk, such as some ligands in DCs and receptors in NK cells [28, 36]. The results demonstrated that MICA/B expression was significantly upregulated in DCs treated with LA. Because MICA/B could stimulate natural killer group 2 member D (NKG2D) on the NK cell surface to activate NK cells [37], we speculated that MICA/B could be the key signal in the LA-mediated NK cell activation. LA-loaded DCs with MICA/B monoclonal antibody pretreatment could not activate NK cells, which further confirmed our hypothesis. Meanwhile, the p38 MAPK pathway could be activated by LA among MICA/B-regulating related pathways [12–14]. LA-loaded DCs could not activate NK cells when p38 MAPK of DCs was blocked; thus, activation of p38 MAPK could be the mechanism by which LA upregulated MICA/B expression. Our previous study indicated that peptides could regulate he p38/MAPK pathway to increase MICA/B expression in hepatocellular carcinoma cells [23], whereas the CTL epitope upregulating MICA/B expression on DCs to activate NK cells has not been reported.

To clarify the mechanism of p38 MAPK pathway activation by LA, we further analysed RNA-seq data and found that the molecular functions of immune-related differentially expressed genes included signalling pattern recognition receptor activity, peptide antigen binding, etc. These results suggested that TLRs, playing critical roles as pattern recognition signal receptors in the innate immune response on DC surface [38], might be a direct target for LA to induce NK activation through DCs. RNA-seq-based transcriptome analysis also demonstrated that the expression levels of TLR4 were downregulated most among the TLRs and the downstream signals of TLR4 (*MYD88* and *TRAF*) were moderately upregulated in DCs with LA treatment. It also showed the most stable binding between TLR4 and LA by MOE. In addition, TAK-242 could significantly inhibit the upregulation of Rael (a ligand for mouse NKG2D with a very similar structure to MICA/B) expression in DCs, the increased frequencies of NK cells, and the improved antitumour effects in HLA-A2.1^+^/ECM1^+^ mouse breast cancer cell-bearing mice treated with LA. Therefore, we speculated that LA could be used as a TLR4 agonist to initiate TLR4 intracellular signals to further activate the p38 MAPK pathway [39–41]. YL did not affect NK activation, possibly because the fact that TLR4 with a deep hydrophobic pocket is more likely to bind hydrophobic molecules, such as LPS. It is extremely difficult for YL with a hydrophilic property to bind to TLR4 relative to LA with a hydrophobic property (Additional file 1: Fig. S6d, e) to initiate intracellular signals for NK activation. The increased expressions of MYD88, TRAF6, MICA/B, and p38 phosphorylation level, and the elevated cytotoxicity of NK cells were attenuated even disappeared DCs were pretreated with TAK242, an inhibitor of TLR4, and pulsed with LA. Previous studies demonstrated that the MHC-I/epitope complex could directly activate NK cells via the KIR3DS1 receptor [42]. To further investigate the role of KIR3DS1 signals on NK cell activation, we used an anti-KIR3DS1 antibody to block the KIR3DS1 receptor on NK cells. The results showed that LA-loaded DCs could still activate NK cells (Additional file 1: Fig. S10), indicating that the HLA-A2.1/LA complex might not activate NK cells via the KIR3D receptor. Therefore, LA-activated NK cells through mediating the TLR4-p38 MAPK pathway to upregulate the MICA/B expression on DC surface to further interact specifically with NKG2D on NK cell surface through ligand-receptor coupling for the DC-NK crosstalk.

CTL epitopes of low-molecular weight possess an inferior stability and a short half-life in vivo, making it difficult to induce robust immune responses [43]. In addition, the strategy of utilizing cytokine-induced DCs to prepare CTL epitope-DC vaccines in vitro has a number of disadvantages, such as complicated operation and a long production period [44]. In this study, we found that the use of peptide alone (LA or YL) did not produce significant antitumour effects (Additional file 1: Fig. S8), which may be associated with its rapid degradation in vivo (Fig. 5j). Some strategies have been used to overcome these disadvantages, including introduction of fatty acid ligands [45], substitution of amino acid cleavage sites [46], and sustained-release preparations [47]. Besides, peptide mixed with adjuvants is a rational option to compensate for the immune response deficiency of peptide application alone [48]. ISA is an ideal immune stimulatory adjuvant [49]. We prepared formulations of ISA-51 [50] and LA or YL, which could enhance the stability of epitopes in vivo, to immunize *HLA-A2.1* transgenic mice for the collection of splenic cells to further inoculate into tumour-bearing NOD/SCID mice for immunological reconstitution. LA-ISA/YL-ISA significantly inhibited the formation and proliferation of xenograft tumours and pulmonary metastases. Additionally, LA could spontaneously activate CD8^+^ T and NK cells to infiltrate into tumour tissues to further exert stronger antitumour effects than YL, which was consistent with the in vitro results*.* Moreover, we validated the antitumour effect on HLA-A2.1^+^/ECM1^+^ murine breast cancer cell-bearing mouse model. These results indicated that LA-ISA based on the double-activation of CD8^+^ T and NK cells could elicit a more potent antitumour immune response than traditional tumour vaccines based on CTL epitopes and has a promising clinical application in the future.

Immune toxicity has become an essential consideration of immunotherapy [51]. In the present research, we found that LA-induced specific CTLs had nonimmune toxicity to PBMCs. NK cells, as the main member of innate immune cells, could accurately distinguish between "self" and "nonself", thereby precisely killing tumour cells rather than normal autologous cells. Hence, LA-induced CTLs had nonimmune toxicity to normal cells, such as PBMCs and DCs. Although studies reported that the in vivo application of ISA-51 could cause allergic reactions [52, 53], the assay on *HLA-A2.1* transgenic mice immunized with LA-ISA and immunologically reconstituted NOD/SCID mice injected with splenocytes showed a promising safety and no immune toxicity in the normal tissues or organs.

## Conclusions

**Fig. 8:**
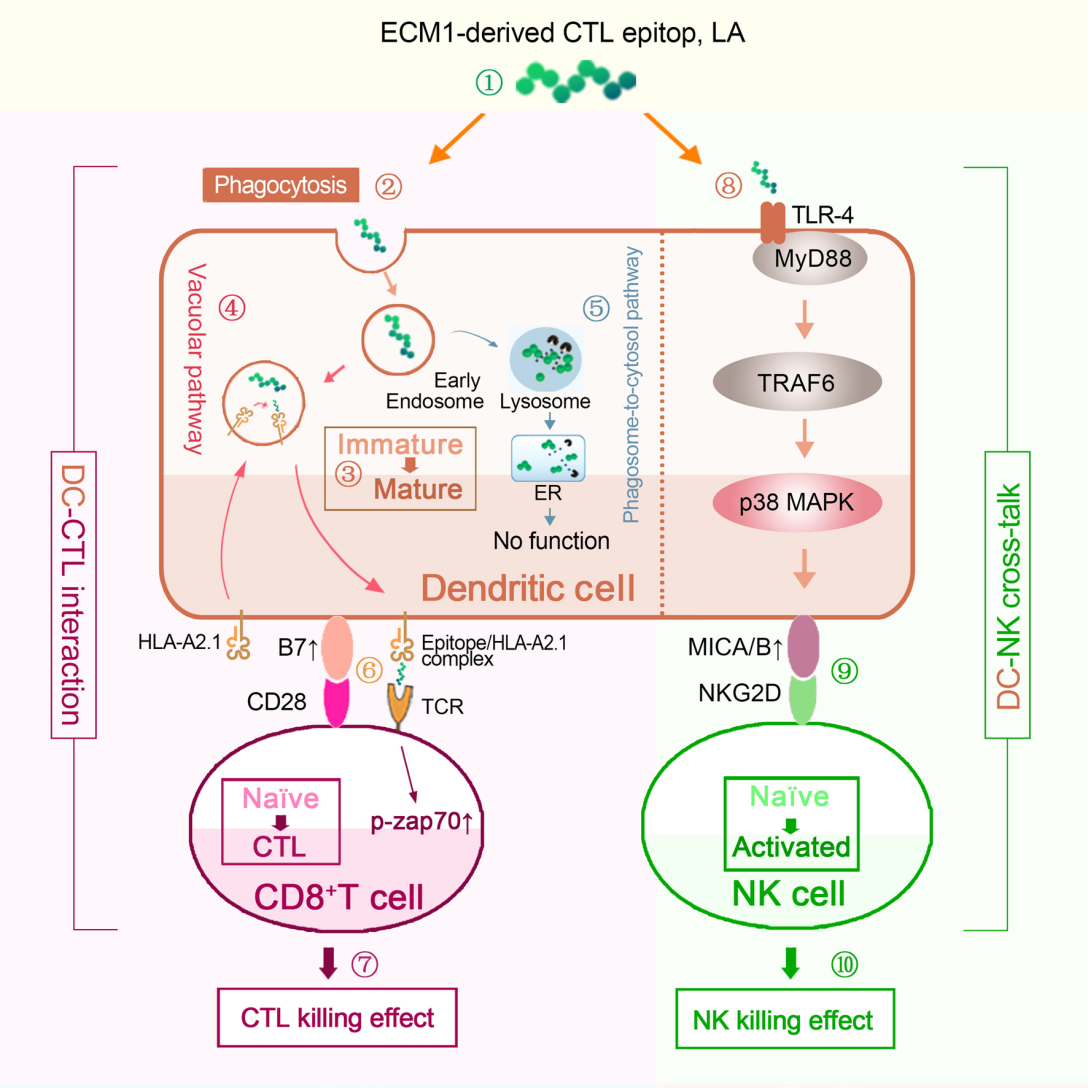
Novel HLA-A2.1-restricted ECM1-derived CTL epitope LA induces a significant DC-CTL and DC-NK antitumour effect. ① Identified novel HLA-A2.1 restricted ECM1-derived CTL epitope LA; ② LA was internalized into DCs mainly through phagocytosis; ③ LA facilitated DC maturation; ④ LA was presented on the surface of DCs via the vacuolar pathway; ⑤ LA passing the phagosome-to-cytosol pathway of DCs was degraded without exerting DC-CTL activation; ⑥ The LA/HLA-A2.1 complex was recognized by the TCR of CD8^+^ T cells. The phosphorylation level of ZAP70 was upregulated, inducing activation of CTL; ⑦ CTL could specifically kill tumour cells (DC-CTL interaction); ⑧ LA-activated TLR4 signal, then activated the p38 MAPK pathway to increase the expression of MICA/B on DCs; ⑨ Upregulated MICA/B expression activated NK cells via MICA/B-NKG2D signal; ⑩ LA enhanced NK antitumour effects (DC-NK crosstalk)

In summary, a novel HLA-A2.1-restricted ECM1-derived epitope LA was presented with double-activation of DC-CTLs and DC-NKs, which could induce a stronger antitumour effect than the traditional CTL epitopes (e.g. YL). We further confirmed that LA could be cross-presented on DC surface in form of the LA/MHC-I complex through the vacuolar pathway for CD8^+^ T cells priming to induce antitumour effect of CTLs. We also demonstrated that activation of NK cells was induced by the LA-mediated TLR4-p38 MAPK pathway upregulating MICA/B expression on DC surface to further activate NKG2D on NK cells through DC-NK crosstalk (Fig. 8). In addition, using xenograft mouse models and an allograft tumour-bearing *HLA-A2.1* transgenic mouse model, we confirmed the double antitumour effect and safety of LA. Our findings highlighted a promising clinical application of LA with double-activation of DC-CTLs and DC-NK cells and laid an experimental foundation for developing a therapeutic tumour vaccine.

## Supplementary Information

**Additional file 1: Figure S1**. Quality confirmation of the peptides. **Figure S2**. Elevated expression of ECM1 in multiple tumours. **Figure S3**. Cytotoxicity of YL/DC-CTLs, LA/DC-CTLs, or LA/DC-NK cells against tumour cell lines. **Figure S4**. Cytotoxicity of YL/DC-CTLs, LA/DC-CTLs, or LA/DC-NK cells against primary tumour cells from HLA-A2.1+/ECM1+ patients. **Figure S5**. No cytotoxicity of LA- or YL-induced DC-CTLs or DC-NK cells on immune cells. **Figure S6**. ECM1-LA/DCs induce the activation of NK cells through the TLR4-p38-MICA/B pathway. **Figure S7**. The frequency of CD4+ T cells in splenocytes (n=3). **Figure S8**. There was no significant antitumour effect in the LA alone- or YL alone-treated group. **Figure S9**. No significant difference was detected in pathological examination of vital organs in xenograft mouse model. **Figure S10**. The KIR3DS1-activating pathway is not involved in the activation of NK cells by LA-pulsed DCs. **Table S1**. HLA-A alleles of tumour (or epithelium) cell lines. **Table S2**. Significant association of ECM1 mRNA expression with the malignant phenotype of various tumours. **Table S3**. Significant association of ECM1 protein expression with the malignant phenotype of various tumours. **Table S4**. Routine blood indexes were examined in different immune groups of HLA-A2.1 transgenic mice. **Table S5**. Main organ indexes are evaluated in different groups of HLA-A2.1 transgenic mice. Supplementary materials and methods.

## Data Availability

All data generated or analysed during the current study are available from the corresponding author on a reasonable request. The gene expression data and corresponding clinical data can be downloaded from The Cancer Genome Atlas (TCGA) database.

## Abbreviations

CTL: Cytotoxic T lymphocyte
DC: Dendritic cell
ECM1: Extracellular matrix protein 1
HLA: Human leukocyte antigen
H&E: Haematoxylin and eosin
IEDB: Immune Epitope Database
IFN-γ: Interferon γ
MHC-I: Major histocompatibility complex class I
MyD88: Myeloid differentiation factor 88
LDH: Lactate dehydrogenase
MICA/B: MHC class I chain-related A/B
MOE: Molecular operating environment
NK: Natural killer
NP: Negative peptide
NS: Normal saline
OD: Optical density
PBMC: Peripheral blood mononuclear cell
Q-TOF: Quadrupole-time-of-flight
SD: Standard deviation
TAA: Tumour-associated antigens
TAP: Transporter associated with antigen processing
TCGA: The cancer genome atlas
TCR: T cell receptor
TRAF6: TNF receptor-associated factor 6
UPLC: Ultra-performance liquid chromatography
WB: Western blot
