# Correction to: HLA-A2.1-restricted ECM1-derived epitope LA through DC cross-activation priming CD8+ T and NK cells: a novel therapeutic tumour vaccine

**DOI:** 10.1186/s13045-021-01176-1

**Published:** 2021-09-29

**Authors:** Zhaojin Yu, Wensi Liu, Ying He, Mingli Sun, Jiankun Yu, Xue Jiao, Qiang Han, Haichao Tang, Bing Zhang, Yunkai Xian, Jing Qi, Jing Gong, Wang Xin, Gang Shi, Fengping Shan, Rui Zhang, Jianping Li, Minjie Wei

**Affiliations:** 1 Department of Pharmacology, School of Pharmacy, China Medical University, No. 13, Beihai Road, Dadong District, Shenyang, Liaoning Province China; 2 Liaoning Key Laboratory of Molecular Targeted Antitumour Drug Development and Evaluation, Liaoning Cancer Immune Peptide Drug Engineering Technology Research Centre, Key Laboratory of Precision Diagnosis and Treatment of Gastrointestinal Tumours, Ministry of Education, China Medical University, No.77, Puhe Road, Shenyang North New Area, Shenyang, Liaoning Province China; 3 Liaoning Medical Diagnosis and Treatment R&D Centre Co. Ltd., Shenyang, Liaoning Province China; 4 The Third Department of Medical Oncology, The Fourth Hospital of China Medical University, Shenyang City, Liaoning Province China; 5 Department of Colorectal Surgery, Cancer Hospital of China Medical University, Liaoning Cancer Hospital & Institute, No.77, Xiaoheyan Road, Dadong District, Shenyang, Liaoning Province China; 6 Department of Immunology, College of Basic Medical Science, China Medical University, Shenyang, Liaoning Province China; 7 Transfusion Medicine Institute, Liaoning Blood Centre, Shenyang, Liaoning Province China; 8 Transfusion Medicine Institute, Harbin Blood Centre, Harbin, Heilongjiang Province China; 9 Department of Pharmacy, Harrison International Peace Hospital, Hengshui, Hebei Province China

## Correction to: J Hematol Oncol (2021) 14:71 https://doi.org/10.1186/s13045-021-01081-7

The original article [1] contains errors in Figure 3i, Figure 6c and Figure S9:

1. In Fig. 3i, the legends of the line chart were marked incorrectly.

**Fig. 3:**
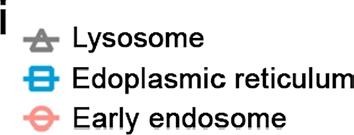
**i** Immunofluorescence analysis of co-localization between LA (green, FITC) and different subcellular compartments (red, early endosome, lysosome or endoplasmic reticulum) in immature DCs (blue, DAPI, nuclear) (n = 3)
2. In Fig. 6c, the panel of ISA CD8a^+^ group was unintentionally duplicated onto the panel of YL-ISA NK1.1^+^ group.

**Fig. 6:**
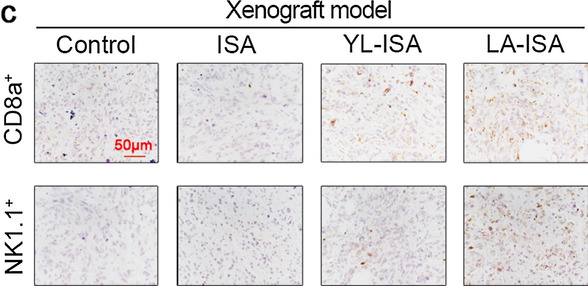
**c** Representative immunohistochemical staining of CD8a and NK1.1 in subcutaneously transplanted tumour tissues
3. In Figure S9, the panel of LA-ISA Liver group was misused.

The corrected figures are presented ahead, and the changes do not affect the conclusions of the paper.

## Supplementary Information

**Additional file 1.**
