# Supplementary material for: HLA-A2.1-restricted ECM1-derived epitope LA through DC cross-activation priming CD8+ T and NK cells: a novel therapeutic tumour vaccine
Source: J Hematol Oncol. 2021 Apr 28;14:71. doi: 10.1186/s13045-021-01081-7 (PMC8082934; doi:10.1186/s13045-021-01081-7)
Supplement: Supplementary file 1 — Additional file 1: Figure S1. Quality confirmation of the peptides. Figure S2. Elevated expression of ECM1 in multiple tumours. Figure S3. Cytotoxicity of YL/DC-CTLs, LA/DC-CTLs, or LA/DC-NK cells against tumour cell lines. Figure S4. Cytotoxicity of YL/DC-CTLs, LA/DC-CTLs, or LA/DC-NK cells against primary tumour cells from HLA-A2.1+/ECM1+ patients. Figure S5. No cytotoxicity of LA- or YL-induced DC-CTLs or DC-NK cells on immune cells. Figure S6. ECM1-LA/DCs induce the activation of NK cells through the TLR4-p38-MICA/B pathway. Figure S7. The frequency of CD4+ T cells in splenocytes (n=3). Figure S8. There was no significant antitumour effect in the LA alone- or YL alone-treated group. Figure S9. No significant difference was detected in pathological examination of vital organs in xenograft mouse model. Figure S10. The KIR3DS1-activating pathway is not involved in the activation of NK cells by LA-pulsed DCs. Table S1. HLA-A alleles of tumour (or epithelium) cell lines. Table S2. Significant association of ECM1 mRNA expression with the malignant phenotype of various tumours. Table S3. Significant association of ECM1 protein expression with the malignant phenotype of various tumours. Table S4. Routine blood indexes were examined in different immune groups of HLA-A2.1 transgenic mice. Table S5. Main organ indexes are evaluated in different groups of HLA-A2.1 transgenic mice. Supplementary materials and methods. [file 13045_2021_1081_MOESM1_ESM.docx]

**
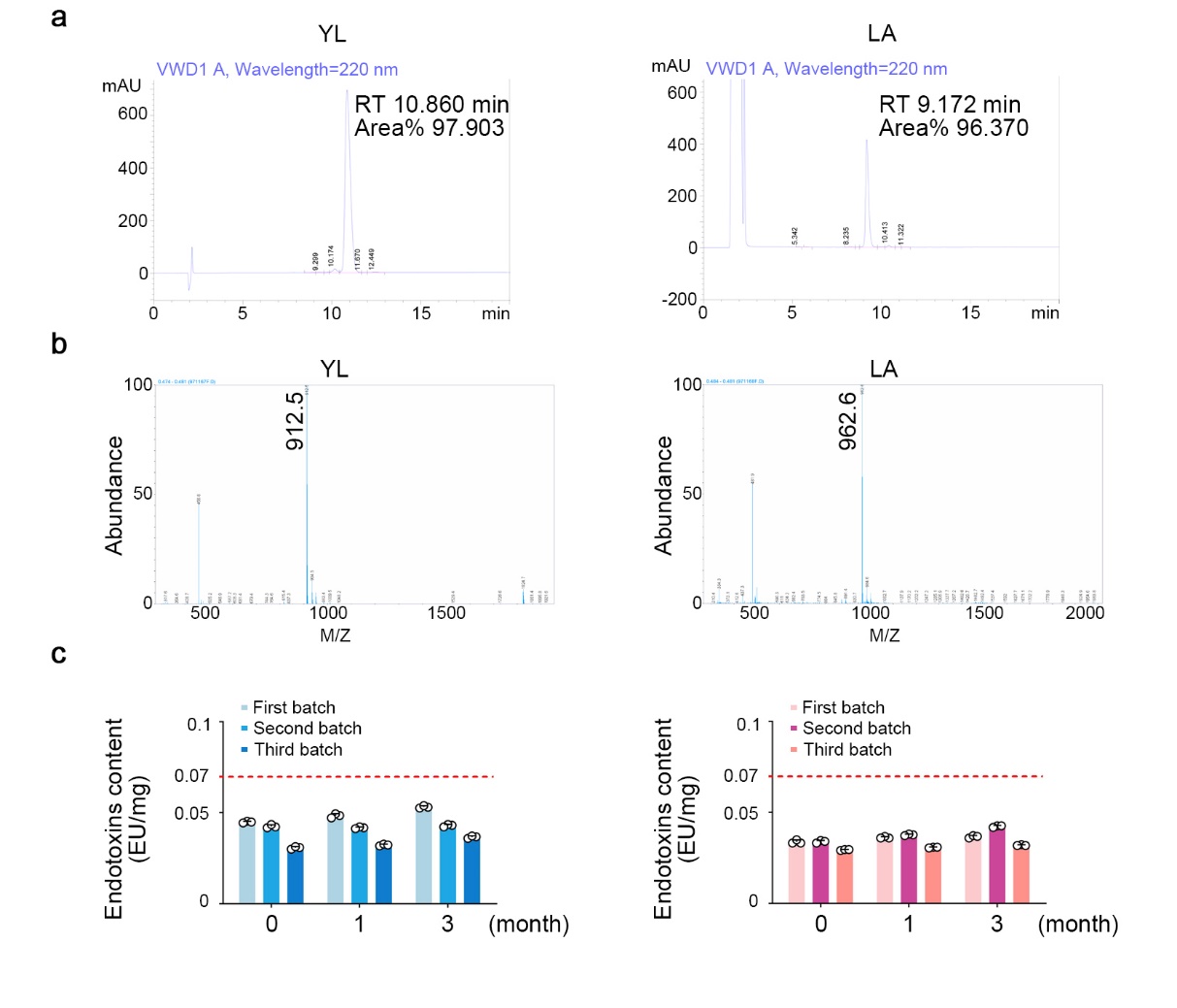
**

**Figure S1. Quality confirmation of the peptides. a,** The purities of YL and LA were both above 95% by HPLC. **b,** The molecular weight of peptides was verified by mass spectrometry. **c,** The endotoxin contents of 3 representative batches of peptides at 3 storage time points (0, 1, 3 months) were detected with limulus amebocyte lysate assay (photometric method).


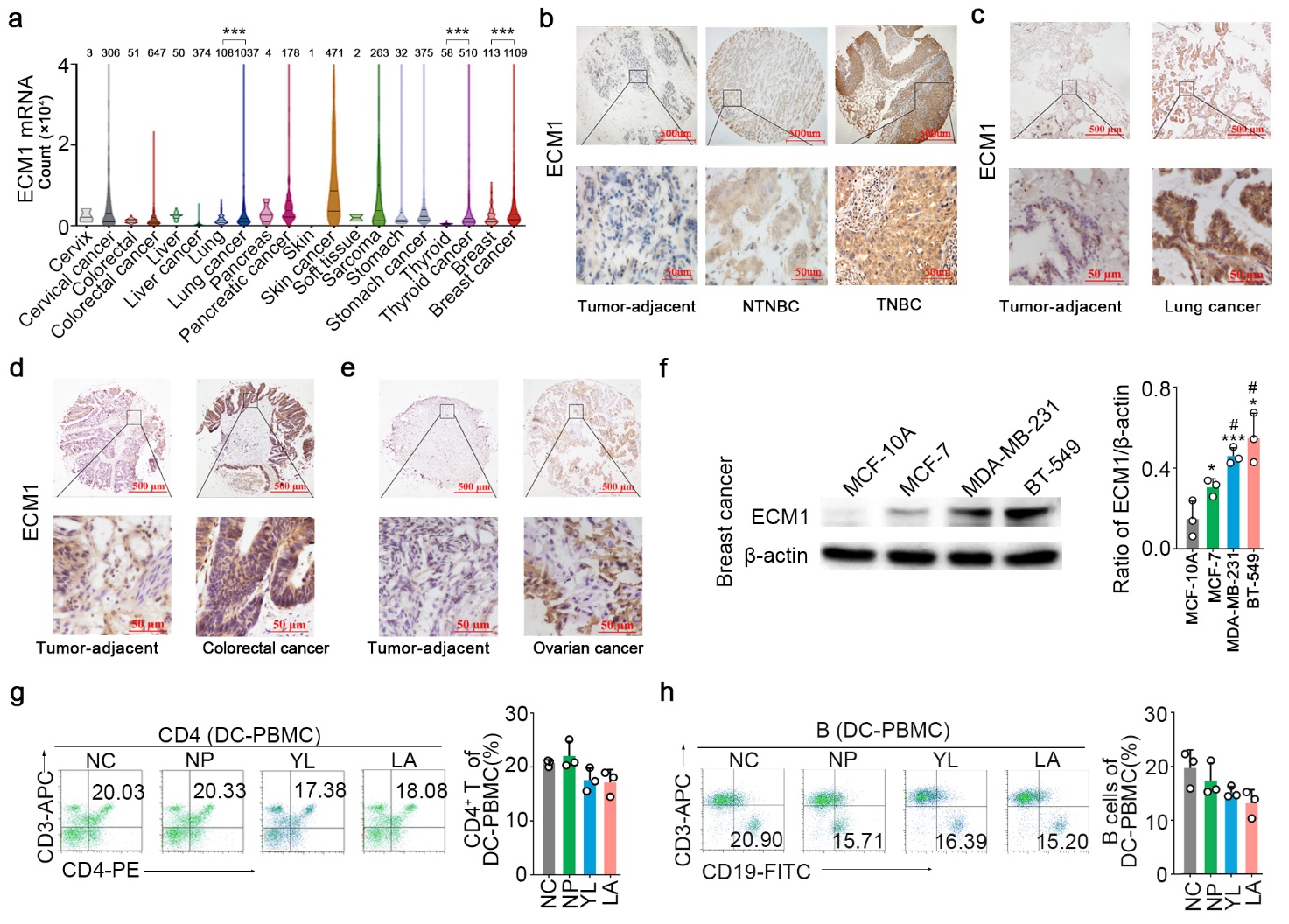
**Figure S2. Elevated expression of ECM1 in multiple tumours. a,** ECM1 mRNA expression was increased in many tumours based on TCGA, such as lung cancer, breast cancer, thyroid cancer et al. *P* values were obtained with Wilcoxon signed ranks test; Error bars denote medians and interquartile ranges; ^***^*P*< 0.001. **b-e,** ECM1 protein expression was increased in many tumours using immunohistochemistry. Scale bars, upper panel, 100 μm, lower panel, 50 μm. **b,** Breast cancer. **c,** Lung cancer. **d,** Colorectal cancer. **e,** Ovarian cancer. **f,** ECM1 protein expression was increased significantly in breast cancer cell lines (MCF-7, MDA-MB-231 and BT-549) using western blot, compared with breast epithelia cell line MCF-10A (*n*=3). *P* values were obtained from independent-samples t-test; error bars denote standard deviation (SD). Compared with MCF-10A, ^*^*P*< 0.05, ^***^*P*< 0.001; compared with MCF-7, ^#^*P*< 0.05. **g, h,** Frequencies of CD4^+^ T and B cells in PBMCs (DC-) stimulated with YL, LA, NP, or NC pulsed DCs (*n*=3).


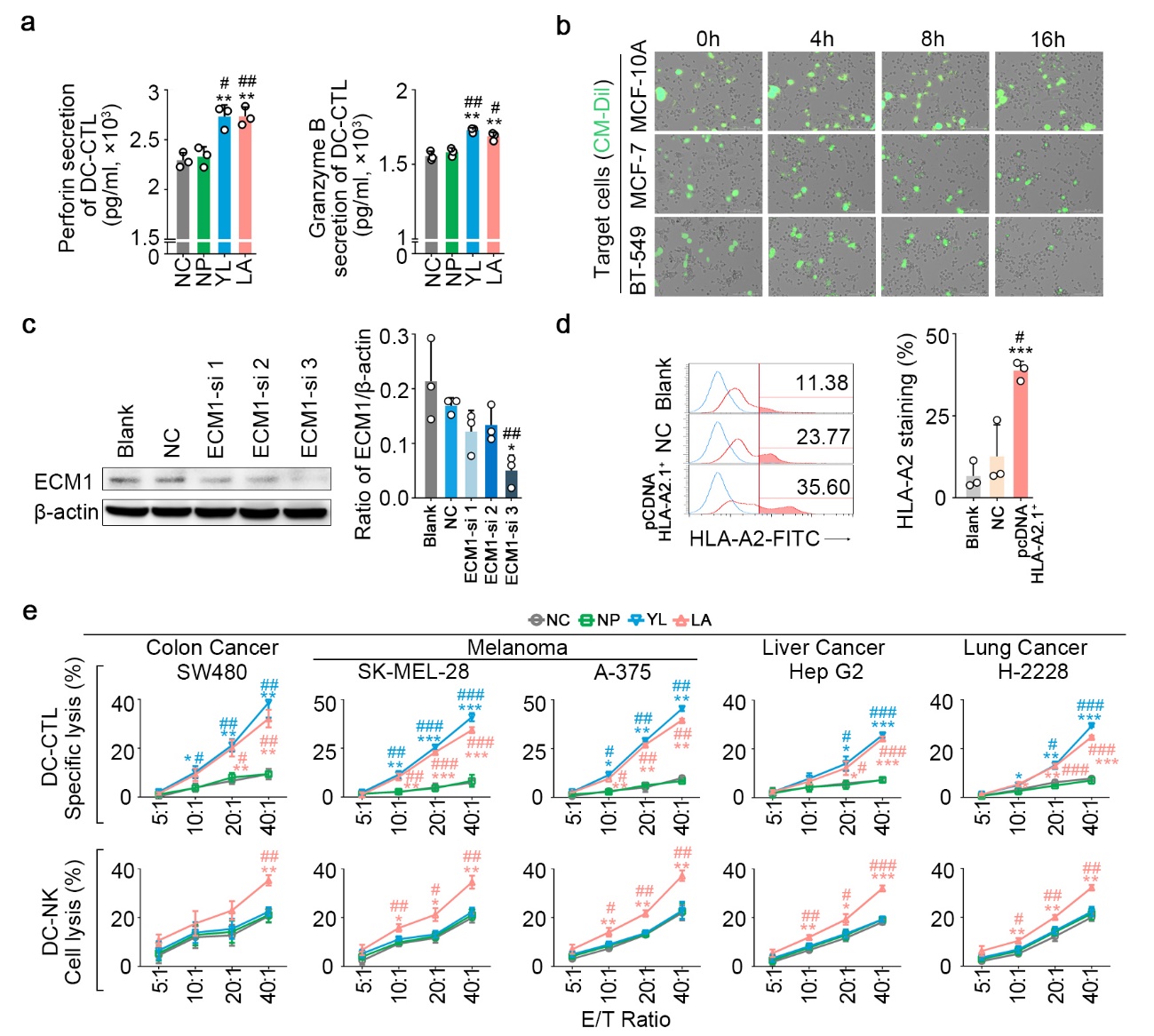
**Figure S3. Cytotoxicity of YL/DC-CTLs, LA/DC-CTLs or LA/DC-NK cells against tumour cell lines. a,** The secretion of perforin/granzyme-B was upregulated in YL/DC-CTL and LA/DC-CTL group using ELISA (*n*=3). **b,** Cytotoxicity of LA/DC-CTLs on breast epithelial cell line MCF-10A, breast cancer cell lines MCF-7 and BT-549 was detected by live-cell imaging. (E/T ratio, 15:1; green, target cells). **c,** The transfection efficiency of siRNA demonstrated that ECM1-si3 could down-regulate protein expression of ECM1 in MDA-MB-231 cells (*n*=3). **d,** Confirmation of vector-mediated HLA-A2.1 overexpression in MCF-7 by flow cytometry (*n*=3). **e,** Cytotoxicity of YL/DC-CTLs, LA/DC-CTLs or LA/DC-NK cells was detected against colon cancer cell line, melanoma cell lines, liver cancer cell line, and lung cancer cell line using calcein-release (*n*=3). **(a, c-e)** *P*-value obtained from independent-samples t-test; error bars denote standard deviation (SD). **(a, e)** Compared with NC, ^*^*P*< 0.05, ^**^*P*< 0.01 ^***^*P*< 0.001; compared with NP, ^#^*P*< 0.05, ^##^*P*< 0.01, ^###^*P*< 0.001. **NC,** negative control, water. **NP,** negative peptide, the peptide (PPGRPSPDN) derived from ECM1 with largest predicted IC_50_ of affinity with HLA-A2.1. **(c, d)** Compared with Blank, ^*^*P*< 0.05, ^***^*P*< 0.001; compared with NC, ^#^*P*< 0.05, ^##^*P*< 0.01. **NC,** negative control, treated with control siRNA or control cDNA.


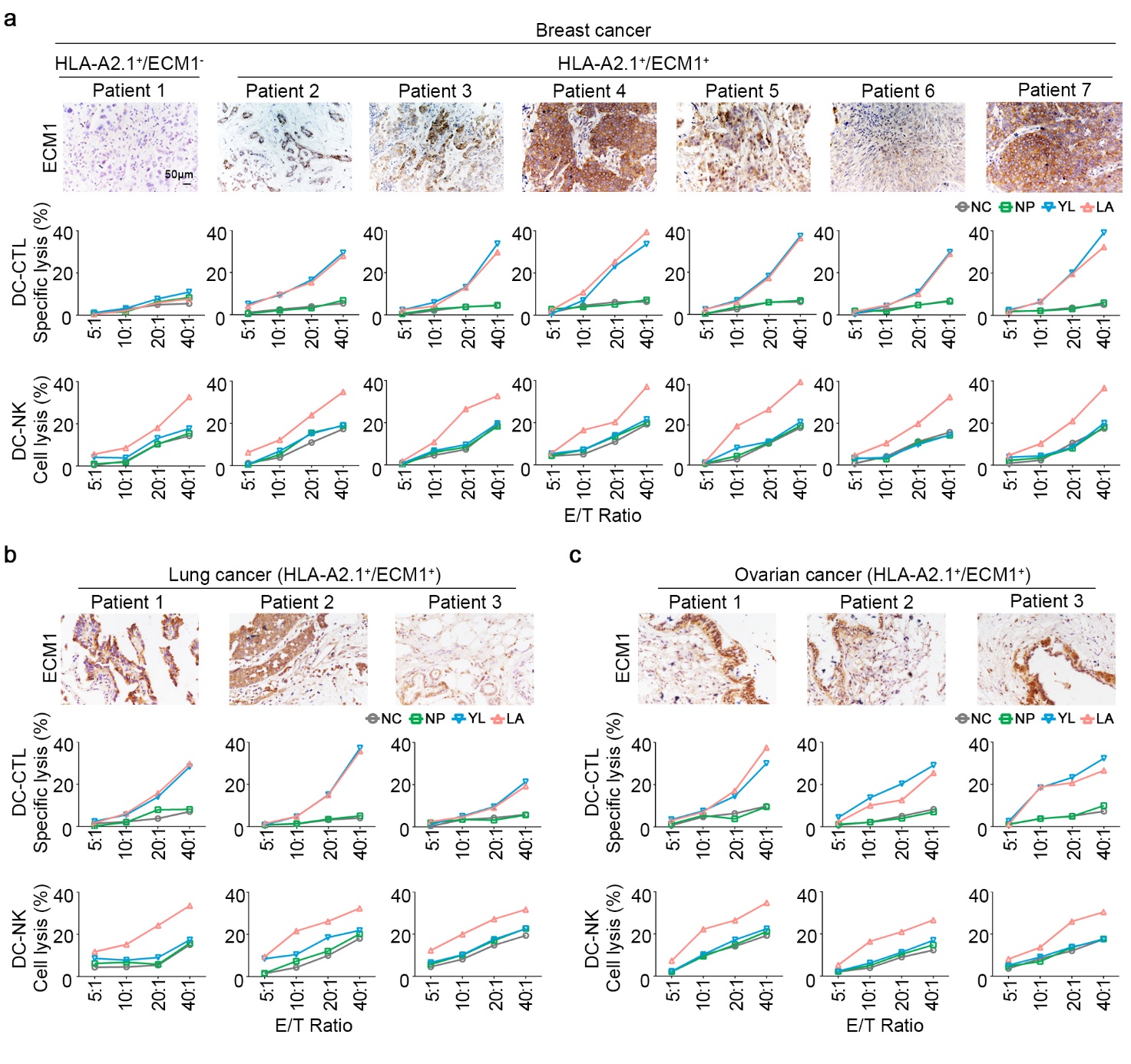
**Figure S4. Cytotoxicity of YL/DC-CTLs, LA/DC-CTLs or LA/DC-NK cells against primary tumour cells from *HLA-A2.1*^+^ patients.** ECM1 expression in tumour tissues was detected by immunohistochemistry. Scale bars, 50 μm. The cytotoxicity of YL/DC-CTLs, LA/DC-CTLs or LA/DC-NK cells was detected against primary tumour cells using calcein-release (*n*=1). **a,** Breast cancer. **b,** Lung cancer. **c,** Ovarian cancer.

**
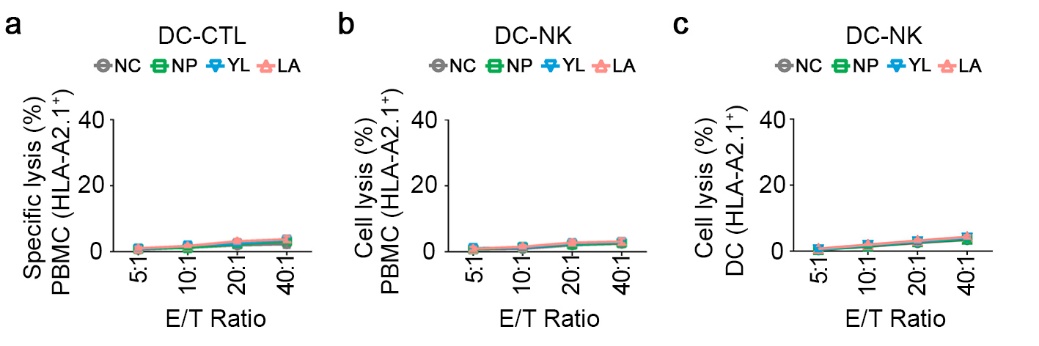
**

**Figure S5 No noticeable cytotoxicity of LA- or YL-induced DC-CTLs or DC-NK cells on immune cells. a,** No noticeable cytotoxicity of LA- or YL-induced DC-CTLs on PBMCs. **b,** No noticeable cytotoxicity of LA- or YL-induced DC-NK cells on PBMCs. **c,** No noticeable cytotoxicity of LA- or YL-induced DC-NK cells on DCs.


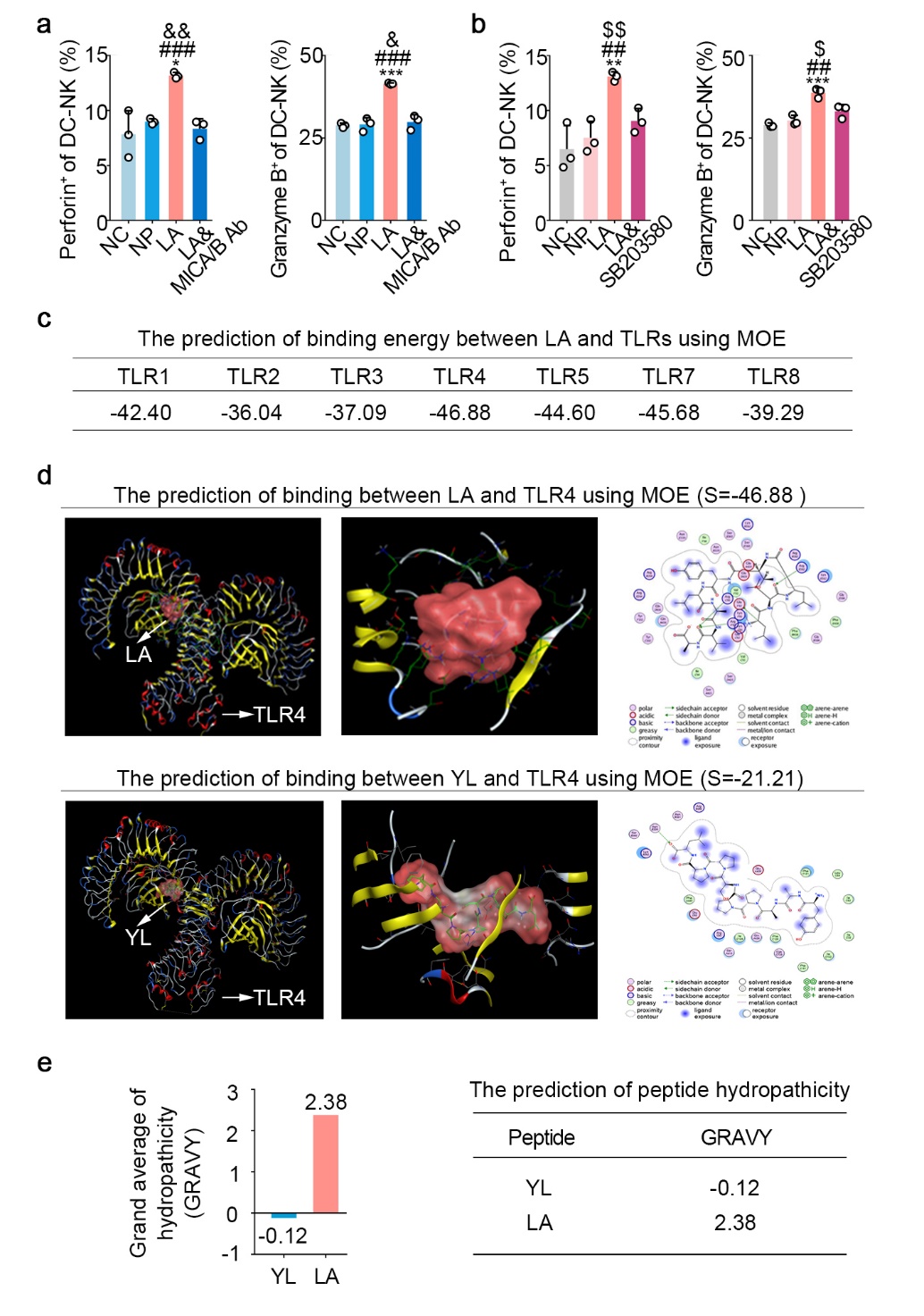
**Figure S6. ECM1-LA/DCs induce the activation of NK cells through the TLR4-p38-MICA/B pathway.** **a,** The perforin and granzyme-B expression of NK cells were significantly increased with stimulation of LA/DC, and MICA/B antibody (Ab) could inhibit the increased expression of perforin and granzyme-B (*n*=3). **b,** SB203580 (10 μM), a inhibitor of p38 MAPK pathway, could block LA/DC-mediated perforin and granzyme-B expression of NK cells (*n*=3). **c,** The combination between LA and TLRs using MOE. The number indicated S value (The free energy between docking molecules). **d,** Interactions between LA (or YL) and the TLR4 molecule. **e,** Prediction of peptide hydropathicity using Novopro (https://www.novopro.cn/tools/calc_peptide_property.html). GRAVY, grand average of hydropathicity. **(a, b)** *P* values were obtained with independent-samples t-test; error bars denote standard deviation (SD). Compared with NC, ^*^*P*< 0.05, ^**^*P*< 0.01, ^***^*P*< 0.001; compared with NP, ^##^*P*< 0.01, ^###^*P*< 0.001; compared with LA&MICA/B Ab, ^&^*P*< 0.05, ^&&^*P*< 0.01; compared with LA&SB203580, ^$^*P*< 0.05, ^$$^*P*< 0.01.


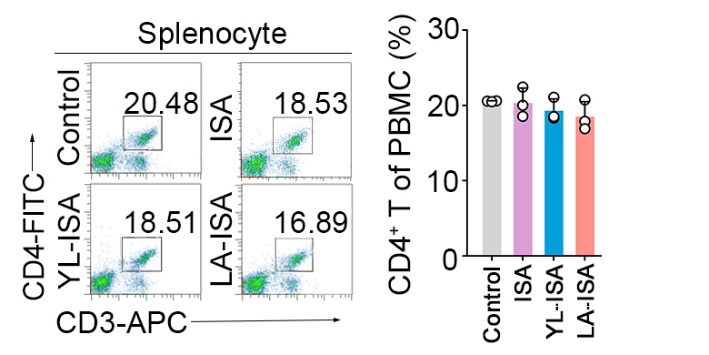


**Figure S7. The frequency of CD4^+^ T cells in splenocytes (*n*=3).**


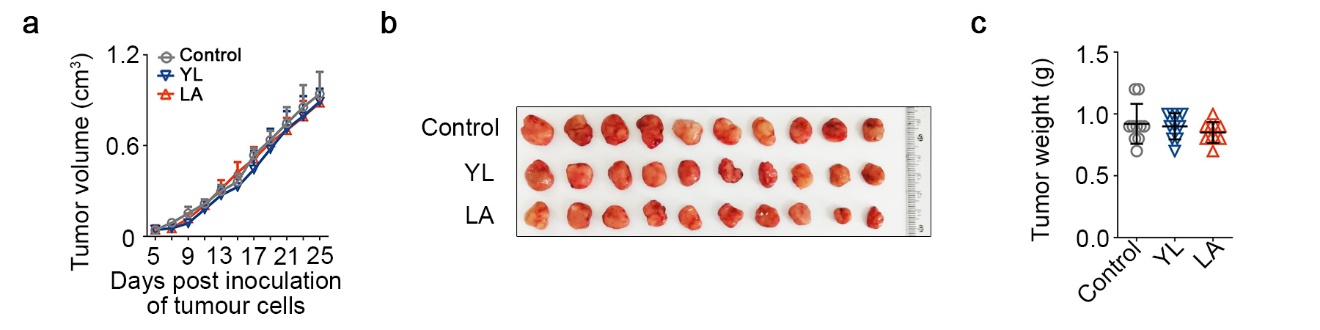


**Figure S8. There was no significant antitumour effect in the LA alone- or YL alone-treated group.** No significant antitumour effect in tumour-bearing NOD/SCID mice inoculated with splenocytes (*n*=10). The splenocytes were harvested from *HLA-A2.1* transgenic mice which were subcutaneously immunized with YL, LA or normal saline (NS). **a,** Growth curve of transplanted tumour. **b,** Transplanted tumour tissues were stripped at day 25 after tumour cell inoculation. **c,** Weight of transplanted tumours at day 25. **Control,** the NOD/SCID mice inoculated with splenocytes from *HLA-A2.1* transgenic mice that were treated with normal saline. (**a, c)** standard deviation (SD).


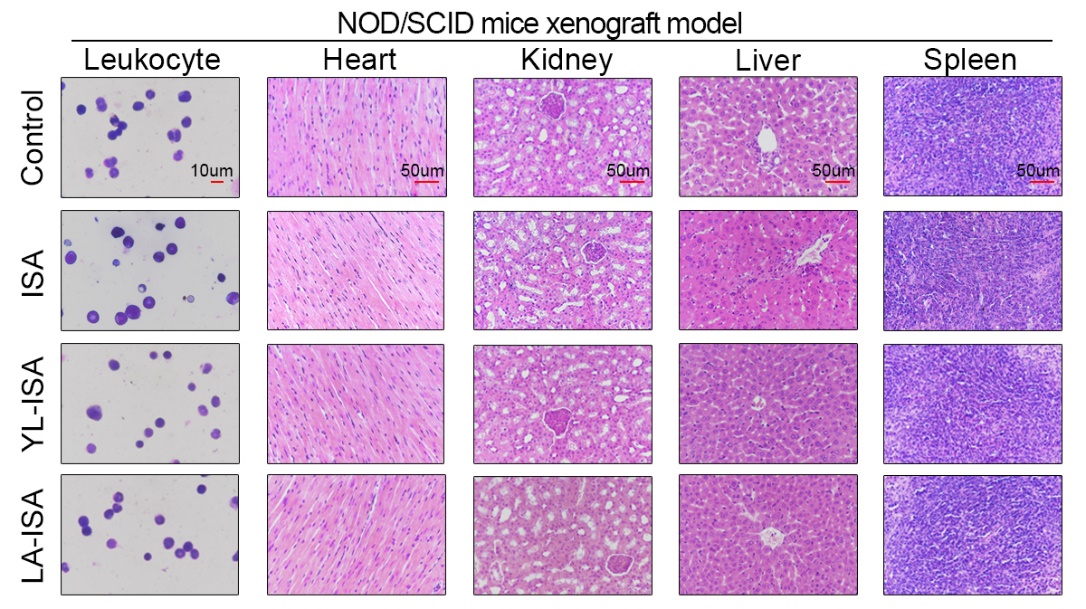


**Figure S9. No significant difference was detected in pathological examination of vital organs in xenograft mouse model.** Leukocyte (Scale bars, 10 μm). Heart, kidney, liver, spleen (Scale bars, 50 μm). **Control,** the NOD/SCID mice inoculated with splenocytes from *HLA-A2.1* transgenic mice treated with normal saline. **ISA,** the NOD/SCID mice inoculated with splenocytes from *HLA-A2.1* transgenic mice treated with ISA. **YL-ISA,** the NOD/SCID mice inoculated with splenocytes from *HLA-A2.1* transgenic mice treated with YL-ISA. **LA-ISA,** the NOD/SCID mice inoculated with splenocytes from *HLA-A2.1* transgenic mice treated with LA-ISA.

**
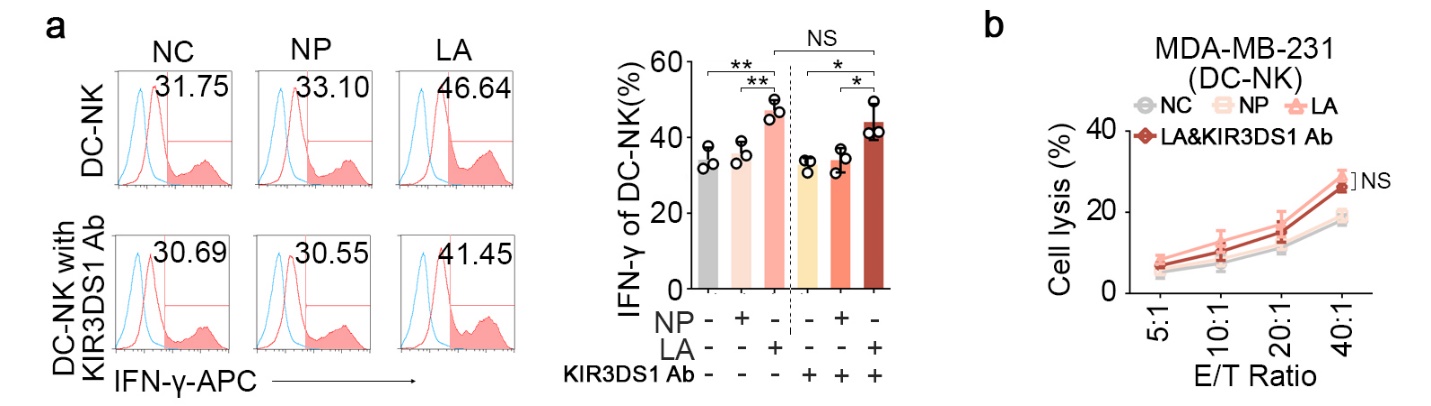
**

**Figure S10. The KIR3DS1-activating pathway is not involved in the activation of NK cells by LA-pulsed DCs. a,** No significant difference of the LA-enhanced IFN-γ level between NK cells and KIR3DS1-antitbody-pretreated NK cells (*n*=3). **b,** No significant difference of the LA-enhanced cell lysis between NK cells and KIR3DS1-antitbody-pretreated NK cells (*n*=3). **NC,** negative control, water. **NP,** negative peptide, the peptide (PPGRPSPDN) derived from ECM1 with largest predicted IC_50_ of affinity with HLA-A2.1. *P* values were obtained from independent-samples t-test; error bars denote standard deviation (SD). ^*^*P*< 0.05, ^**^*P*< 0.01.

**Table S1. *HLA-A* alleles of tumour (or epithelium) cell lines**

| Cell line | Position | Benign or malignant | Genotype of HLA-A | |
| --- | --- | --- | --- | --- |
| MCF-7 | Breast | Malignant | 2402 | 6802 |
| MDA-MB-231 | Breast | Malignant | 0201 | 0217 |
| BT-549 | Breast | Malignant | 0201 | 0101 |
| MCF-10a | Breast | Benign | 0101 | 0201 |
| H2228 | Lung | Malignant | 0201 | 0301 |
| Hep G2 | Liver | Malignant | 0201 | 2402 |
| SW480 | Colon | Malignant | 0201 | 2402 |
| SK-MEL-28 | Skin | Malignant | 0101 | 0201 |
| A-375 | Skin | Malignant | 0101 | 0201 |

**Table S2. Significant association of ECM1 mRNA expression with malignant phenotype in various tumours.**

| Tumour type | Feature | Categorie | ECM1 | | *P* ^a^ |
| --- | --- | --- | --- | --- | --- |
|  |  |  | Low(%) | High(%) |  |
| Cervix cancer | Outcome state | Alive | 124(53.2) | 109(46.8) | **0.042** |
|  |  | Dead | 28(39.4) | 43(60.6) |  |
|  | Tobacco smoking history | ≤1 | 81(56.3) | 63(43.8) | **0.021** |
|  |  | >1 | 49(41.9) | 68(58.1) |  |
|  | Total number of pregnancies | ≤3 | 83(57.2) | 62(42.8) | **0.040** |
|  |  | >3 | 53(44.5) | 66(55.5) |  |
| Colorectal cancer | T stage | 1-2 | 73(58.4) | 52(41.6) | **0.041** |
|  |  | 3-4 | 237(48.2) | 255 (51.8) |  |
|  | N stage | N0 | 188(53.6) | 163(46.4) | 0.064 |
|  |  | N1-2 | 122(46.0) | 143(54.0) |  |
| Pancreatic cancer | T stage | 1-2 | 19(61.3) | 12(38.7) | 0.088 |
|  |  | 3-4 | 64(44.4) | 80(55.6) |  |
| Skin cancer | T stage | 0-2 | 69(48.3) | 74(51.7) | 0.076 |
|  |  | 3-4 | 94(39.0) | 147(61.0) |  |
| Thyroid cancer | T stage | 1-2 | 169(55.0) | 138(45.0) | **0.004** |
|  |  | 3-4 | 81(42.0) | 112(58.0) |  |
|  | Stage | 1 | 151(53.7) | 130(46.3) | **0.046** |
|  |  | 2-4 | 98(44.7) | 121(55.3) |  |
| Breast cancer | N stage | 0 | 277(53.9) | 237(46.1) | **0.010** |
|  |  | 1-3 | 256(46.0) | 300(54.0) |  |

^a^ *P*-value obtained from Pearson Chi-Square test or Fisher's Exact Test.

**Table S3. Significant association of ECM1 protein expression with malignant phenotype of various tumours.**

| Tumour type | Feature | Categorie | ECM1 | | *P* ^b^ |
| --- | --- | --- | --- | --- | --- |
|  |  |  | Low(%) | High(%) |  |
| Breast cancer | Age | <50 | 58(33.9) | 113(66.1) | **0.022** |
|  |  | ≥50 | 30(22.1) | 106(77.9) |  |
|  | Tumour type | NTNBC | 86(32.8) | 176(67.2) | **<0.001** |
|  |  | TNBC | 2(4.4) | 43(95.6) |  |
|  | Histological stade | Ⅰ~Ⅱa | 56(33.3) | 112(66.7) | **0.047** |
|  |  | Ⅱb~Ⅳ | 32(23.0) | 107(77.0) |  |
|  | Nodes metastasis | 0 | 70(34.0) | 136(66.0) | **0.003** |
|  |  | 1 | 18(17.8) | 83(82.2) |  |
| Colorectal cancer | CA19-9(0-39) | Low | 26(81.3) | 6(18.8) | **0.028** |
|  |  | High | 19(55.9) | 15(44.1) |  |
|  | Death | No | 22(75.9) | 7(24.1) | **0.040** |
|  |  | Yes | 15(50.0) | 15(50.0) |  |
| Lung cancer | Age(years) | ≤60 | 20(95.2) | 1(4.8) | **0.038** |
|  |  | >60 | 12(70.6) | 5(9.4) |  |
|  | N stage | ≤N0 | 22(95.7) | 1(4.3) | **0.013** |
|  |  | >N0 | 9(64.3) | 5(35.7) |  |
|  | Stage | ≤Ⅰ | 20(95.2) | 1(4.8) | **0.023** |
|  |  | >Ⅰ | 10(66.7) | 5(33.3) |  |

^b^ *P*-value obtained from Pearson Chi-Square test or Fisher's Exact Test.

**Table S4. Routine blood indexes are examined in different groups of *HLA-A2.1* transgenic mice.**

|  | Control | ISA | YL-ISA | LA-ISA |
| --- | --- | --- | --- | --- |
| WBC (10^9^/L) | 230.2±4.8 | 228.5±4.02 | 229.25±1.69 | 229.93±2.26 |
| Lymph# (10^9^/L) | 9.57±0.84 | 9.35±0.65 | 9.45±0.18 | 9.55±0.45 |
| Mid# (10^9^/L) | 18.88±0.86 | 18.18±0.86 | 18.39±0.44 | 18.58±0.63 |
| Gran# (10^9^/L) | 201.78±3.13 | 201.3±2.08 | 201.6±1.16 | 201.8±1.25 |
| Lymph% | 4.15±0.26 | 4.1±0.22 | 4.12±0.05 | 4.15±0.16 |
| Mid% | 8.18±0.22 | 7.95±0.24 | 8.07±0.19 | 8.08±0.2 |
| Gran% | 88±0.36 | 87.95±0.46 | 87.87±0.17 | 87.77±0.35 |
| HGB (g/L) | 128.17±13.87 | 127.17±10.53 | 121.17±10.67 | 118.33±23.73 |
| RBC (10^12^/L) | 2.43±0.32 | 2.38±0.22 | 2.23±0.44 | 2.22±0.47 |
| HCT (%) | 11.22±1.36 | 11.1±0.95 | 10.68±1.81 | 10.12±2.08 |
| MCV (fL) | 46.92±0.99 | 46.97±1.67 | 46.17±1.14 | 45.92±0.51^*^ |
| MCH (pg) | 53±1.69 | 53.3±1.07 | 53.31±0.88 | 53.37±1.16 |
| MCHC (g/L) | 1144.17±33.55 | 1142.17±24.59 | 1159.17±21.72 | 1170.83±20.22^*^ |
| RDW-CV (%) | 13.87±0.37 | 14.5±1.13 | 14.27±1.38 | 14.07±0.35 |
| RDW-SD (fL) | 23.43±0.75 | 24.07±2.05 | 23.97±1.94 | 23.7±0.42 |
| PLT (10^9^/L) | 127.17±29.12 | 132.67±20.34 | 127.17±33.33 | 125.33±27.27 |
| MPV (fL) | 7.88±0.26 | 7.7±0.27 | 7.72±0.15 | 7.63±0.18 |
| PDW | 14.7±0.31 | 14.57±0.24 | 14.56±0.27 | 14.5±0.19 |
| PCT (%) | 0.1±0.02 | 0.1±0.02 | 0.11±0.02 | 0.1±0.02 |

^*^Compared with control group, *P*<0.05, *n*=6. **Control,** the *HLA-A2.1* transgenic mice treated with normal saline. **WBC,** white blood cell; **Mid,** intermediate cell; **Gran,** granulocyte; **HGB**, hemoglobin; **RBC,** red blood cell; **HCT,** hematocrit; **MCV,** mean corpuscular volume; **MCH,** mean corpuscular hemoglobin; **MCHC,** mean corpuscular hemoglobin concentration; **RDW-CV,** red cell distribution width-coefficient of variation; **RDW-SD,** red cell distribution width-standard deviation; **PLT,** platelet; **MPV**, mean platelet volume; **PDW**, platelet distribution width; **PCT**, plateletocrit.

**Table S5. Main organs indexes are evaluated in different groups of *HLA-A2.1* transgenic mice.**

|  | Control | ISA | YL-ISA | LA-ISA |
| --- | --- | --- | --- | --- |
| Weight (g) | 21.65±5.1849 | 22.3±3.4583 | 22.9±3.2243 | 21.95±4.2908 |
| %Heart index | 0.0069±0.0015 | 0.0070±0.0003 | 0.0070±0.0006 | 0.0071±0.0010 |
| %Liver index | 0.047±0.0071 | 0.051±0.0056 | 0.052±0.0084 | 0.050±0.0063 |
| %Spleen index | 0.0040±0.0003 | 0.0045±0.0005 | 0.0046±0.0003 | 0.0046±0.0006 |
| %Renal index | 0.012±0.0019 | 0.012±0.0009 | 0.012±0.0011 | 0.012±0.0011 |
| %Lung index | 0.0089±0.0016 | 0.0093±0.0004 | 0.0085±0.0006 | 0.0093±0.0014 |
| %Thoracic gland index | 0.0052±0.0014 | 0.0052±0.0008 | 0.0054±0.0007 | 0.0053±0.0011 |
| %Brain index | 0.027±0.0064 | 0.026±0.0034 | 0.026±0.0033 | 0.026±0.0051 |

*n*=6. Control, the *HLA-A2.1* transgenic mice treated with normal saline.

**Supplementary materials and methods**

**ECM1 mRNA expression analysis based on TCGA database**

The data of gene expression and clinical information were extracted from TCGA dataset (<http://cancergenome.nih.gov/>), and the expression data between cancer tissues and adjacent tissues were compared. ECM1 expression level was divided into the high expression group and the low expression group based on the median value to identify the prognosis in tumour patients.

**Immunohistochemical staining**

Tissue microarrays (BRN801B, BR8018, BC081120a, BR487c, alenabio, China) were purchased including 81 normal or neighboring noncancerous tissues, 262 NTNBC tissues, and 45 TNBC tissues. 43 lung cancer tissues and 35 para-carcinoma tissues were obtained from the Department of Thoracic Surgery, the First Affiliated Hospital of China Medical University in 2015. The intestinal tissues were collected from patients in the Department of Gastrointestinal Surgery, the Fourth Affiliated Hospital of China Medical University between 2008 and 2011, including 92 cancer tissues and 81 para-cancerous tissues. 48 ovarian carcinoma tissues and 46 adjacent normal tissues were obtained from Department of Gynecology, Shengjing Hospital, China Medical University between 2011 and 2015. All tissues were embedded in paraffin to establish tissue sections or the tissue microarray for immunohistochemistry. The sections were deparaffinized, rehydrated and covered in sodium citrate buffer (pH=6.0) for antigen retrieval. Then the slides were treated with hydrogen peroxide and goat serum. The sections were incubated with primary antibody (1:100 dilution; ab126629; abcam) overnight at 4 °C. Subsequently, The sections were incubated with biotinylated goat anti-rabbit IgG and streptavidin horseradish peroxidase (KIT-0100R, MXB Biotechnologies). After being stained with 3,3-diaminobenzidine (DAB), slides were counterstained with hematoxylin, dehydrated and mounted. As a negative control, the primary antibody was replaced PBS.

**Western blot analysis of ECM1 expression**

Proteins were extracted with the whole Protein Extraction Kit (BB-3101, BestBio, China) and quantified using the BCA protein assays. Equal amount of proteins (20 μg) was resolved on 12% (v/v) SDS-polyacrylamide gel electrophoresis (SDS-PAGE), and then transferred to a PVDF membrane. The membrane was blocked with 2% (w/v) BSA and then incubated with the primary antibody (rabbit anti-human ECM1 polyclonal antibody, 1:1000, ab126629, abcam; rabbit anti-human β-actin polyclonal antibody, 1:1000, 20536-1-AP, Proteintech) overnight at 4 °C. Subsequently, goat anti-rabbit HRP-conjugated secondary antibody (1:10000 dilution, E030120, EarthOx, USA) was incubated with the membrane for 1 h at room temperature. The stripe was visualized with ECL detection system (Pierce, Thermo Fisher Scientific, USA) and densitometry was performed using ImageJ (1.8.0).

**Analysis of protein-peptide interactions**

We downloaded the crystal structure of HLA-A2.1 molecule (PDB ID: 4K7F) from RCSB PDB database (http://www.rcsb.org/). The HLA-A2.1 molecule was preprocessed with QuickPrep and docked with ECM1-derived peptides or negative peptide using MOE Dock (2018 package, Chemical Computing Group, Montreal, QC, Canada).

**ECM1 epitopes binding and complex stability assay**

Affinity of ECM1 epitopes was examined by HLA-A2 binding assay. T_2_ cells (1×10^6^ per well) were cultured with peptide (50 μg/mL) and β2-microglobulin (3 μg/ml, M4890, SIGMA) for 18 h.

For complex stability, the T_2_ cells were subsequently cultured in serum-free IMDM containing Brefeldin A (10 μg/mL, HY-16592, MedChem Express) for 1 h. Then, the cells were cultured for 0, 2, 4, 6 or 18 h at 37 °C with 5% CO_2_, respectively.

HLA‐A2.1 expression was detected by flow cytometry using FITC anti-human HLA-A2 (343304, Biolegend) and was quantified using fluorescence index (FI). FI = (epitope peptide mean fluorescence intensity - background mean fluorescence intensity)/background mean fluorescence intensity. FI＞1.5, the strong binding affinity. DC_50_, the time required for 50% reduction of the mean fluorescence intensity value at 0 h. DC_50_>6h, the high stability.

**Collection of PBMC**

PBMCs from whole blood of *HLA-A2.1*^+^ healthy volunteers (The detection of HLA-A alleles assisted by Beijing Bo Furui Gene Diagnosis Technology, Co,. Ltd) were obtained by density gradient centrifugation using lymphocyte separation medium (human, 7111011, Dakewe).

**Generation of monocyte-derived DCs**

Monocytes were obtained from PBMC using monocyte attachment medium (C-28051, PromoCell), and were cultured in DC generation medium (C21050, PromoCell). The phenotype of DC was identified by flow cytometry with PE anti-human CD11c (301605, BioLegend) and APC anti-human HLA-DR (307609, BioLegend) staining, PE anti-human CD80 (305208, BioLegend), PE anti-human CD86 (374206, BioLegend) and FITC anti-human CD83 (305305, biolegen).

**ELISPOT assay for IFN-γ secretion**

The PBMC were cultured in serum-free hematopoietic cell medium (X-VIVO^TM^ 15, Lonza). IL-2 (10 ng/ml) was added on day 1, day 4, day 7. On day 1 and day 7, the recombinant ECM1 protein (10 μg/mL) was added. PBMCs were stimulated with the candidate epitope peptides on day 12, respectively. Human IFN-γ precoated ELISPOT kit (2110005, Dakewe) was performed on day 13 according to the instruction of the manual. Spots were counted using an ELISPOT image analyzer (Bioreader4000, Biosys).

Epitope/DC-CTL and epitope/DC-NK were generated as described in Materials and Methods. The epitope peptide-pulsed DCs as stimulators were added on day 12 to perform ELISPOT assay.

Immune cells from *HLA-A2.1*^+^ healthy volunteers were divided into different groups (blank, DCs, CD8^+^ T cells, and DCs&CD8^+^ T cells; blank, DCs, NK cells, and DCs&NK cells). The cells were stimulated with YL or LA on day 1 and day 6, and were triggered with YL or LA on day 12 to perform Elispot assay.

**Phenotypic analysis of PBMC (DC-) stimulated with epitope-pulsed DCs**

PBMCs (DC^-^) were stimulated with epitope peptide-loaded DCs at a ratio of 20: 1 on day 1 and day 6, and were obtained on day 12. Phenotypes of PBMC were detected by flow cytometry. The frequencies of CD4^+^ and CD8^+^ T cells in PBMCs was analyzed using FITC anti-human CD4 (317408, BioLegend), PE anti-human CD8a (300908, BioLegend), and APC anti-human CD3 (300311, BioLegend). The frequency of NK cells (CD3^-^CD16^+^CD56^+^) was labeled with APC anti-human CD56 (318309, BioLegend), PE anti- Human CD16 (302008, BioLegend) and FITC anti-human CD3 (300406, BioLegend). APC anti-human CD3 and FITC anti-human CD19 (302205, BioLegend) were used to detect the frequency of B cells.

**CFSE proliferation assay**

During the induction of epitope/DC-CTL or epitope/DC-NK as described in Materials and Methods, epitope/DC-CTL cells or epitope/DC-NK cells were cultured with CFSE (5 μM, C375, Dojindo) for 10 minutes on day 0. After washing, the CFSE-labeled cells were measured by flow cytometry on day 8.

**Phenotypic analysis of epitope/DC-CTL**

The epitope/DC-CTL cells were analyzed by flow cytometry using FITC anti-human CD45RO (304204, BioLegend) and PE/Cy5 anti-human CD69 (310908, BioLegend).

**Analysis of intracellular cytokine expression**

Epitope/DC-CTL cells were generated as described in Materials and Methods. The epitope/DC-CTL cells were cocultured with *HLA-A2.1*^+^/ECM1^+^ tumour cells overnight on day 12, then stimulated with Cell Activation Cocktail (423303, BioLegend) for 4-6 h. The cells were fixed and were washed with permeabilization buffer. The cells were analyzed by ﬂow cytometry using APC anti-human Perforin (308111, BioLegend), FITC anti-human/mouse Granzyme B Recombinant (372205, BioLegend), PE anti-human CD178 (306407, BioLegend).

**Analysis of perforin and granzyme B secretion by ELISA**

Epitope/DC-CTL cells were generated as described in Materials and Methods. The epitope/DC-CTL cells were cocultured with HLA-A2.1^+^/ECM1^+^ tumour cells overnight on day 12. The supernatant was collected to perform the ELISA assay (Human perforin ELISA Kit, 1118302, Dakewe; Human Granzyme B ELISA Kit, 1118502, Dakewe) according to the manufacturer's instructions.

**Cell transfection**

ECM1-specific siRNAs (target sequence: ECM1-si 1, GAACGTGGCTCTAGTGTCT; ECM1-si 2, GGAGGATACCCTTGACAAA; ECM1-si 3, GCTTCAACATCAATTATCT) were purchased from Guangzhou RiboBio Co., Ltd. *HLA-A2.1* cDNA and control cDNA were purchased from Shanghai Genechem Co., Ltd. 100 nM ECM1-specific siRNAs or control siRNA was transfected into MDA-MB-231 cells according to the manufacturer's instructions (lipofectamine 2000, 11668019, Invitrogen). 2000ng *HLA-A2.1* cDNA or control cDNA was transfected into MCF-7 cells. Cells were harvested 72 h later for further experiments.

**Isolation and culture of primary cells**

Human breast cancer tissues, lung cancer tissues, and ovarian cancer tissues (within 4 hours in vitro) were cut into pieces of 1 mm^3^. The pieces were cultured in D-Hanks solution containing collagenase IV (1 mg/ml) and hyaluronic acid (0.15 mg/mL) at 37 °C for 6 h with shaking. The detection of HLA-A alleles assisted by Beijing Bo Furui Gene Diagnosis Technology, Co,. Ltd. Primary tumour cells were harvested from the precipitate using 0.25% trypsin-EDTA in a 37 °C water bath for 5 min, and then pre-cooled HF (1 g/L glucose, 2% FBS D-Hanks) was added. Cells were cultured in primary cell culture medium (DMEM/F12, 12500062, gibco; bFGF 20 μg/L, GF446, Sigma-Aldrich; hEGF 20 μg/L, E9644, Sigma-Aldrich; hydrocortisone 5 mg/L, G8450, Solarbio; β-estradiol 5 mg/L, E8140, Solarbio; insulin, 10 mg/L, I8830, Solarbio; 5% FBS; 1% Penicillin-Streptomycin).

**Tumour histoculture end-point staining computer-image-analysis**

Human breast cancer tissues were cut into pieces of 1 mm^3^ (one piece per well) and were cultured overnight. The light projection image of the tissue block was collected by tissue activity tester (H20041368, GuangZhou Handy Biotechnology CO.,LTD), and the area of each tissue block (area, A) was determined. Then epitiope/DC-CTL cells were cocultured with the tumour tissue block for 3 days, respectively. Then MTT were added for a 3-h incubation, and the blue formazan crystals were formed. The area (blue area, BA) of each tissue block was determined. Calculate inhibition rate (IR%)=[1-(BA_treated_/A_treated_)/(BA_control_/A_control_)]×100%.

**Scanning electron microscopy of DC morphology**

Differently stimulated DCs were seeded on poly-lysine-coated slides, were fixed with 2.5% glutaraldehyde, were dehydrated with ethanol, were frozen at -80 ℃, and were dried for 1 h. The scanning electron microscopy was used to observe the morphology of DCs by Shanghai Bingxin Biotechnology Co., Ltd.

**FITC-dextran uptake assay**

After incubation at 4 °C for 30 min (precooling), differently stimulated DCs were divided two groups to incubate at 4 °C or 37 °C for 1 h. Meanwhile, FITC-dextran (0.5 mg/ml, FD40S, Sigma) was added to the medium. The mixture was subsequently incubated at 4 °C for 30 min and washed 5 times. Flow cytometry was used to detect the mean fluorescence intensity of DCs.

**Analysis of IL‐12p70 secretion by ELISA**

IL‐12p70 secretion by differently stimulated DCs was examined according to the manufacturer's instructions (Human IL-12p70 ELISA Kit, 1111202, Dakewe).

**LA uptake by DCs**

LA-FITC (50 μg/mL) was added to DCs and incubated for 12 h at 4°C or 37°C. Flow cytometry was used to detect the mean fluorescence intensity of DCs.

**LA internalization by DCs**

Clathrin-mediated endocytosis inhibitor chlorpromazine (5 μg/ml, HY-B0407A, MedChemExpress), phagocytosis inhibitor cytochalasin D (10 μg/ml, B1176, biovision), macropinocytic inhibitor amiloride (5.32 μg/ml, HY-B0285, MedChem Express), receptor-mediated internalization inhibitor heparin (25 μg/ml, B3602, ApexBio), scavenger receptor-mediated internalization inhibitor Poly (I:C) (50 μl/ml, 31852-29-6, Invivogen), mannose receptor-mediated internalization inhibitor CD206 antibody (10 μg/ml, 321101, Biolegend) were used to pre-treat DCs for 30 min at 37 °C. LA-FITC (50 μg/mL) was added to DCs and incubated for 12 h at 37°C. Flow cytometry was used to detect the mean fluorescence intensity of DCs.

**Immunofluorescent analysis of LA localization in DCs**

DCs were incubated with LA-FITC (50 μg/mL) at 37 °C for 15 min, 30 min, 1 h, 2 h, and 3 h, respectively. Then the DCs were fixed. On the one hand, the cells were cultured with mouse anti-human EEA-1 monoclonal antibody (1:500, sc-137130, Santa) or rabbit anti-human calreticulin monoclonal antibody (1:400, 12238, CST) overnight at 4°C, and were subsequently cultured with Alexa Fluor-568 goat anti-mouse IgG H&L (1:500, ab175473, abcam) or Alexa Fluor-568 goat anti-rabbit IgG H&L (1:1000, ab175471, abcam) for 1 h on next day. On the other hand, the DCs were cultured with CellLight^TM^ Lysosomes-RFP, BacMam 2.0 (1 μL/10^4^ cell, C10504, Invitrogen) for 16 h. The above DCs were observed by confocal microscope analyses. Lasers at 405 nm, 488 nm, and 543 nm excited the fluorescence of DAPI, FITC and Alexa 568, respectively. Manders overlap coefficients were calculated using NIS-Elements AR 4.40.00 software to quantify colocation level between LA and different subcellular compartments (early endosomes, lysosomes, or endoplasmic reticulum).

**Trace analysis of LA inside DCs**

DCs were pre-treated with molecule surface trafficking inhibitor primaquine (50 μM, HY-12651, MedChem Express), vesicle secretion inhibitor brefeldin A (10 μg/ml), or TAP-inhibiting proteins ICP47 (10 μg/ml, CSB-YP360979HWY, CUSABIO) for 30 min, and were incubated with LA (50 μg/mL) overnight at 37 °C. Then DCs were co-cultured with PBMC (DC-) at a ratio of 1: 20. After 72 h incubation, FITC anti-human CD3, PE anti-human CD8 and APC anti-human IFN-γ were used to detect the expression of IFN-γ (502512, BioLegend) in CD8^+^ T cells by flow cytometry.

**Isolation of cytoplasmic membrane protein**

DCs were incubated with different epitopes (50 μg/mL) overnight. The cytoplasmic membrane protein isolation kit (SM-005, Invent) was used to extract the plasma membrane proteins from DCs for UPLC-Q-TOF analysis based on the manufacturer's instruction.

**Ultra Performance Liquid Chromatography**

Chromatographic analysis was performed on Acquity UPLC system (Waters Ltd., Elstree, U.K.). Cytoplasmic membrane protein samples were injected into a C 18 BEH column (100 mm×2.1 mm, 1.7 μm; Waters) at 30 °C with a low rate of 300 μL/min. The mobile phase A contains acetonitrile and 0.1% formic acid. The mobile phase B blends 0.1% formic acid with H_2_O. The injection volume was 5 μL. A linear gradient elution program was applied as follows: 70-15 % B from 0.00 to 12.00 min, 15-70% B from 12.00 to 12.1 min, and hold at 70 % B for 2 min.

**Epitope Profiling by UPLC-Quadrupole-Time-of-Flight (Q-TOF) Mass Spectrometry**

After separation by UPLC, mass spectrometry was performed using a Q-TOF Premier (Waters, Manchester, U.K.) and a Xevo G2-XS Q-TOF with an electrospray ionization (ESI) source (Waters, Manchester, UK) for global peptide profiling. MS parameters were set with capillary voltage at 2.5 kV, cone voltage at 30 V, source temperature at 120 °C, desolvation temperature at 400 °C, desolvation gas flow at 800 L/h, and cone gas flow at 20 L/h based on positive ion-mode. The scanrange was from m/z 100 to 1500.

**Analysis of ZAP70 phosphorylation**

Epitope/DC-CTL cells were generated as described in Materials and Methods. Epitope/DC-CTL cells were stimulated with NP-, YL-, or LA-loaded DCs on day 12. Subsequently, cells were collected at 0 min, 10 min, 30 min, and 1 h, respectively. Western blot analysis was performed using Phospho-Zap-70 (Tyr319) /Syk (Tyr352) Rabbit mAb (1:1000, #2717, CST) and Zap-70 Rabbit mAb (1: 2000, #3165, CST).

**RNA seq**

Oxford Nanopore Technologies Long Read Processing. Raw reads were first filtered with minimum average read quality score=7 and minimum read length=500bp. Ribosomal RNA were discarded after mapping to rRNA database. Next, full-length, non-chemiric (FLNC) transcripts were determined by searching for primer at both ends of reads. Clusters of FLNC transcripts were obtained after mapping to reference genome with mimimap2, and consensus isoforms were obtained after polishing within each cluster by pinfish.

Remove redundant. Consensus sequences were mapped to reference genome using minimap2. Mapped reads were further collapsed by cDNA. Cupcake package with min-coverage=85% and min-identity=90%. 5’ difference was not considered when collapsing redundant transcripts.

Find fusion transcript. The criteria for fusion candidates is that a single transcript must: (1) must map to 2 or more loci (2) minimum coverage for each loci is 5% and minimum coverage in bp is ≥ 1 bp (3) total coverage is ≥ 95% (4) distance between the loci is at least 10kb

Structure analysis.Transcripts were validated against known reference transcript annotations with gffcompare. AS events including IR, ES, AD, AA and MEE were identified by the AStalavista tool. SSR of the transcriptome was identified using MISA. APA analysis was conducted with TAPIS. CDS were predicted by TransDecoder.

Transcription factors prediction. Plant transcription factors were identified with iTAK, while Animal transcription factors were identified from animalTFDB.

Gene functional annotation. Gene function was annotated based on the following databases: NR (NCBI non-redundant protein sequences); Pfam (Protein family); KOG/COG/eggNOG (Clusters of Orthologous Groups of proteins); Swiss-Prot (A manually annotated and reviewed protein sequence database); KEGG (Kyoto Encyclopedia of Genes and Genomes); GO (Gene Ontology).

**MICA/B expression analysis**

DCs were incubated with LA (50 μg/mL) at 37 °C overnight. The next day, DCs were detected by flow cytometry using PE anti-human MICA/B (320906, BioLegend). Then we used western blot to detect the expressions of MICA/B. The primary antibody was Rabbit Anti-MICA+MICB antibody (1:1000 dilution, ab224702, abcam).

**Analysis of intracellular cytokine secretion**

The epitope/DC-NK, cocultured with LA-pulsed DCs overnight, were analyzed by intracellular flow cytometry. The antibodies were APC anti-human Perforin, FITC anti-human/mouse Granzyme B Recombinant, APC anti-human IFN-γ (502512, BioLegend).

**Analysis of phosphorylation of ERK, p38, Akt**

DCs were incubated with LA (50 μg/mL) at 37 °C overnight. The expression of ERK, p38, Akt and p-ERK, p-p38 and p-Akt in whole protein were detected by western blot. The primary antibodies were mouse anti-human ERK 1/2 monoclonal antibody (1:1000 dilution, SC-514302, Santa Cruz), rabbit anti-human p38 polyclonal antibody (1:1000 dilution, 9212s, CST), rabbit anti-human Akt monoclonal antibody (1:1000 dilution, 4691s, CST), mouse anti-human p-ERK 1/2 monoclonal antibody (1:1000 dilution, sc-81492, Santa Cruz), rabbit anti-human p-p38 Cloning antibody (1:1000 dilution, 9211s, CST), rabbit anti-human p-Akt monoclonal antibody (1:2000 dilution, 4060s, CST). DCs were pre-treated with SB203580 (p38 inhibitor, 10 μM, A8254, ApexBio) for 1 h in LA&SB203580 group.

**Molecular docking analysis**

The crystal structures of DC surface receptors were obtained from PDB databank (<http://www.rcsb.org>), such as TLR1 (PDB ID:6NIH), TLR2 (PDB ID:5D3I), TLR3 (PDB ID: 5GS0), TLR4 (PDB ID: 4G8A), TLR5 (PDB ID: 3J0A), TLR7 (PDB ID: 5GMF), TLR8 (PDB ID: 3WN4), HLA-A2.1(PDB ID:4K7F). The energy minimization was conducted to remove water molecules, ionic groups, heteroatoms, as well as to protonate the proteins using MOE. The conformational search of E6 was performed to determine the optimal conformations (Top 100) to dock the receptors on frequently activated sites. The results were determined by analyzing the systematic scores and interaction forces.

**Expression of TLR4 signaling pathway-related proteins**

DCs were incubated with LA (50 μg/mL) at 37 °C overnight. The expression levels of MyD88, TRAF6, p-p38, p38, MICA/B, Vinculin in whole protein were detected by western blot. DCs were pre-treated with TAK-242 (inhibitor of TLR4 Signaling, 10 μM, A3850, ApexBio) for 1 h in LA&TAK-242 group. The primary antibodies were rabbit anti-MyD88 antibody (1:1000 dilution, ab219413, abcam), rabbit anti-TRAF6 antibody (1:1000 dilution, ab40675, abcam), Vinculin Monoclonal antibody (1:2000 dilution, [66305-1-Ig](http://www.ptgcn.com/products/Vinculin-Antibody-66305-1-Ig.htm), Proteintech).

**The mechanisms about NK activation via the KIR3D**

Z27 (IgG1, anti-KIR3DS1) is able to bind the extracellular regions of KIR3DS1. NK cells were pre-treated with KIR3DS1-antitbody for 1 h. LA (50 μg/mL) was added to DCs to induce epitope/DC-NK as mentioned above. Epitope/DC-NKs were stained with APC anti-human IFN-γ (502512, BioLegend) and detected by flow cytometry. Epitope/DC-NK-mediated lysis of tumour cells was analyzed by LDH assay.

**In vivo real-time imaging study**

Nude mice (female, 18-22 g, 6-8 weeks, VitalRiver) were subcutaneously injected in the inguinal region with YL-FITC, YL-FITC-ISA, LA-FITC, or LA-FITC-ISA. At 10 min, 30 min, 4 h, 24 h or 72 h after injection, nude mice were performed under general anesthesia with isoflurane (Hebei Yipin Pharmaceutical Co., Ltd.) inhalation, and were observed by a in vivo imaging system (LB983, Berthold) with an excitation wavelength of 485 nm and an emission wavelength of 535 nm.

**Phenotypic analysis of the DCs obtained from the immunized *HLA-A2.1* transgenic mice**

PBMCs were obtained from peripheral blood of *HLA-A2.1* transgenic mice that were vaccinated for 3 times and then were lysed with red blood cell lysis buffer (R1010, Solarbio). PBMCs were stained with APC anti-mouse CD11c (117309, BioLegend), PE anti-mouse CD86 (105007, BioLegend), PE anti-mouse CD80 (104707, BioLegend), and mouse Rae1 pan specific fluorescein-conjugated antibody (FAB17582F, RD). The cells were detected by flow cytometry.

**Phenotypic analysis of the splenocytes obtained from immunized *HLA-A2.1* transgenic mice**

Splenocytes of *HLA-A2.1* transgenic mice were stained with APC anti-mouse CD3 (100236, BioLegend), FITC anti-mouse CD3 (100203, BioLegend), FITC anti-mouse CD4 (100509, BioLegend) and PE-anti-mouse CD8a (100707, BioLegend) to detect CD4^+^ T and CD8^+^ T cell frequency in CD3^+^ T cells by flow cytometry. APC anti-mouse CD49b (108909, biolegen) staining was used to detect the NK cell frequency by flow cytometry. Splenocytes were stained with APC anti-mouse CD3, FITC anti-mouse CD3 (100203, BioLegend), PE anti-mouse CD8a, APC anti mouse-perforin (154304, BioLegend), FITC anti human/mouse Granzyme B Recombinant (372206, BioLegend) to detect perforin and Granzyme B expression levels in CD8^+^ T cells.

**Cell virus infection**

For ECM1 knock-in analysis, lentiviral vectors (GV341) were purchased from Shanghai Genechem Co., Ltd. ECM1 cDNA was cloned into Ubi-MCS-3FLAG-SV40-puromycin. After AgeI/NheI enzyme-cutting and DNA sequencing, the recombinant plasmid (LV-ECM1, 66118-1, Genechem) was constructed successfully. The mouse mammary cancer cell line (E0771) was transfected with encoded ECM1-encoded lentiviral particles (LV-ECM1, MOI=10). The stably transfected cells were selected by culturing in fresh culture medium with puromycin (2 μg/ml). The stable expression of plasmids was confirmed.

For HLA-A2.1 knock-in analysis, lentiviral vectors (CV237) were purchased from Shanghai Genechem Co., Ltd. *HLA-A2.1* cDNA was cloned into Ubi-MCS-IRES-Hygromycin. After XbaI/XbaI enzyme-cutting and DNA sequencing, the recombinant plasmid (LV-HLA-A, 66083-1, Genechem) was constructed successfully. ECM1^+^ E0771 cells were transfected with HLA-A2.1-encoded lentiviral particles (LV-HLA-A2.1, MOI=10). The stably transfected cells were selected by culturing in fresh culture medium with hygromycin B (200 μg/ml). The stable expression of plasmids was confirmed.
