# Supplementary material for: Correction to: HLA-A2.1-restricted ECM1-derived epitope LA through DC cross-activation priming CD8+ T and NK cells: a novel therapeutic tumour vaccine
Source: J Hematol Oncol. 2021 Sep 29;14:158. doi: 10.1186/s13045-021-01176-1 (PMC8482666; doi:10.1186/s13045-021-01176-1)
Supplement: Supplementary file 1 — Additional file 1. [file 13045_2021_1176_MOESM1_ESM.docx]

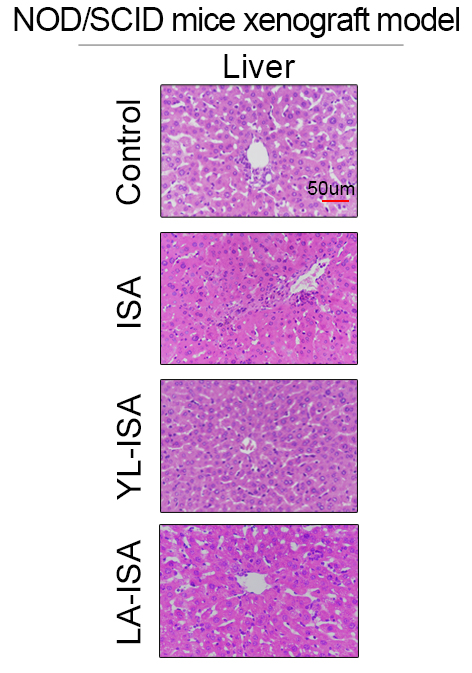


**Figure S9. No significant difference was detected in pathological examination of vital organs in xenograft mouse model.** Liver (Scale bars, 50 μm).
